# Bioprinting Organs—Science or Fiction?—A Review From Students to Students

**DOI:** 10.1002/adhm.202502103

**Published:** 2025-12-03

**Authors:** Nicoletta Murenu, Camilla Mussoni, Mateo S. Andrade Mier, Paula Buettner, Nathaly Chicaiza‐Cabezas, Yi‐Yu Robin Dai, Jessica Faber, Maren Fiedler, Zan Lamberger, Xuen Jen Ng, Vanessa Moessler, Anna Rederer, Jonas Roeder, Sabrina Stecher, Katinka Theis, Jeanette Weigelt, Silvia Budday, Gregor Lang, Natascha Schaefer

**Affiliations:** ^1^ Institute for Clinical Neurobiology University Hospital Würzburg, Julius‐Maximilians‐University Würzburg Versbacherstr. 5 97078 Würzburg Germany; ^2^ Department for Functional Materials in Medicine and Dentistry Institute of Functional Materials and Biofabrication and Bavarian Polymer Institute Julius‐Maximilians‐University Würzburg Pleicherwall 2 97070 Würzburg Germany; ^3^ Department of Mechanical Engineering, Institute of Continuum Mechanics and Biomechanics Friedrich‐Alexander‐University Erlangen‐Nürnberg Dr.‐Mack‐Str. 81 90762 Fürth Germany; ^4^ Experimental Renal and Cardiovascular Research, Department of Nephropathology Institute of Pathology and Department of Cardiology Friedrich‐Alexander‐University Erlangen‐Nürnberg 91054 Erlangen Germany; ^5^ Section for Experimental Oncology and Nanomedicine, Department for Otorhinolaryngology, Head and Neck Surgery University Hospital Erlangen Glücksstrasse 10a 91054 Erlangen Germany; ^6^ Department of Medicine 4, Nephrology and Hypertension University Hospital Erlangen Ulmenweg 18 91054 Erlangen Germany; ^7^ Department of Trauma, Hand, Plastic and Reconstructive Surgery University Hospital Würzburg 97080 Würzburg Germany; ^8^ Department of Gynecology and Obstetrics University Hospital Erlangen Friedrich‐Alexander‐University Erlangen‐Nürnberg Universitätsstr. 21/23 91054 Erlangen Germany; ^9^ Department of Biomaterials University of Bayreuth Wallstraße 4 95445 Bayreuth Germany; ^10^ Institute of Biomaterials Friedrich‐Alexander‐University Erlangen‐Nürnberg Cauerstr. 6 91058 Erlangen Germany

**Keywords:** bioinks, maturation, mechanical testing, organ printing, printability, vascularization

## Abstract

Bioprinting technology has attracted significant attention in the field of tissue engineering, enabling the precise placement of cells, biomaterials, and biomolecules to construct 3D tissue and organ structures. This review explores the feasibility of bioprinting functional organs by assessing current advancements in the field. A poll conducted among people from diverse backgrounds reveals common optimism regarding the future of organ bioprinting and its role in medicine and other fields. The article is conceptualized from a student‐to‐student perspective to provide a brief overview of key aspects of bioprinting, including bioinks, crosslinking techniques, bioprinting methods, and the maturation process required to develop functional tissues. Furthermore, it highlights recent progress in printing specific tissues as models for studying healthy and diseased tissues as well as implantable grafts. While there are still significant challenges that require the integration of technologies from engineering, biomaterials science, cell biology, physics, and medicine, ongoing research continues to address these complexities. The possibilities of bioprinting tissues and organs go beyond minimizing dependence on animal testing and advancing drug discovery; indeed, this approach also opens the door to accessible personalized medicine and presents a viable solution to the worldwide organ donor shortage.

## Graphical abstract

Bioprinting artificial organs has the potential to revolutionize the medical field. This is a comprehensive review of the bioprinting workflow delving into the latest advancements in bioinks, materials and bioprinting techniques, exploring the critical stages of tissue maturation and functionality. This study provides an overview of the progress in each stage and the future potential of functional bioprinted tissues.

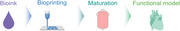

## 1 Introduction: Social Relevance, Potential, and Expectations in Biofabrication

Tissue engineering is a relatively new field that combines living cells, biocompatible materials, and biochemical and physical factors to create tissue‐like structures. Its primary goal is to implant these constructs into the body to repair injuries or replace failing organs, providing an alternative to the ongoing shortage of donor organs. The earliest recorded attempts date back to 3000 B.C., with skin grafting mentioned in Sanskrit texts from India, followed by autologous skin grafting in Europe in 1794.^[^ ^1^ ^]^ Although the field has demonstrated promising results in constructing various tissues—primarily through the use of decellularized organs, such as blood vessels,^[^ ^2^, ^3^, ^4^ ^]^ the heart,^[^ ^5^, ^6^ ^]^ lungs,^[^ ^7^, ^8^ ^]^ liver,^[^ ^9^, ^10^ ^]^ kidneys,^[^ ^11^, ^12^ ^]^ bladder,^[^ ^13^ ^]^ and pancreas^[^ ^14^ ^]^—numerous challenges remain in developing complex, functional tissues that accurately replicate human organ systems.^[^ ^15^ ^]^ Bioprinting, a subset of tissue engineering, has emerged as a promising strategy after over two decades of research.^[^ ^16^ ^]^ This technique uses bioprinters guided by computer‐aided systems (design or manufacturing‐CAD/CAM)^[^ ^17^ ^]^ to print the desired shape and organization of cells^[^ ^18^, ^19^ ^]^ and biomaterials. Bioprinting is defined as “*the use of material transfer processes for patterning and assembling biologically relevant materials, such as cells, with a prescribed organization to accomplish one or more biological functions*”.^[^ ^20^ ^]^

**Figure 1:**
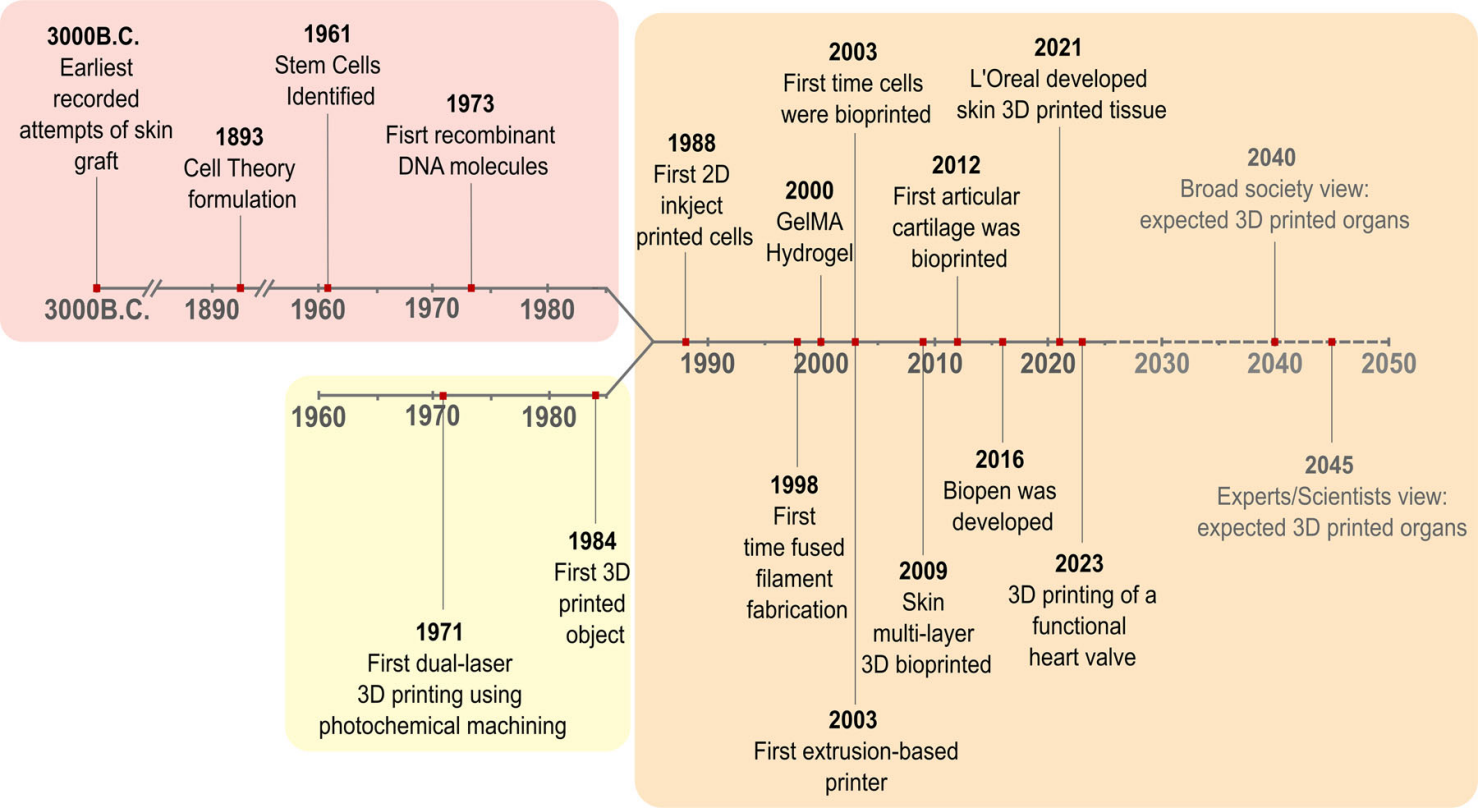
Timeline of various key events in the history of bioprinting, showcasing the progression from early theoretical concepts to recent developments, covering bioink formulations, printing technologies, and preclinical and industrial applications. The scheme highlights the convergence between biology (red) and the field of additive manufacturing (yellow) within biofabrication (orange).

Using those systems, bioprinting can achieve high spatial resolution and functionality in tissue constructs, offering significant advantages over traditional in vitro models. Multimaterial printing, which integrates living cells with supporting materials such as vascular scaffolds, further enhances tissue survival and functionality, enabling the creation of even more complex structures. The evolution of this technology relies on the cell theory of 1890, which recognized the cell as the fundamental structural and functional unit of life, with all organisms being composed of cells and all cells arising from preexisting ones. This insight laid the foundation for tissue engineering, defined as “*the use of physical, chemical, biological, and engineering processes to control and direct the aggregate behavior of cells*”,^[^ ^21^ ^]^ which applies the principle that assembling the right cells can recreate functional tissues and organs. However, it began with the development of 3 dimensional (3D) printing, patented in 1984, which allowed us to print the first 3D objects. Another breakthrough occurred in 1998, when the first fused filament construct was successfully fabricated, paving the way for the burst of extrusion‐based printing that occurred in 2003.^[^ ^22^ ^]^ Since the early 2000s, the field has advanced rapidly. Innovations such as the BioPen have opened new possibilities for surgical repairs. Recent developments in skin and heart valve printing demonstrate the growing potential of this technology, with experts predicting that in the next 20 years,^[^ ^23^ ^]^ bioprinting may provide a viable solution for organ transplantation (**Figure** **1**).

**Figure 2:**
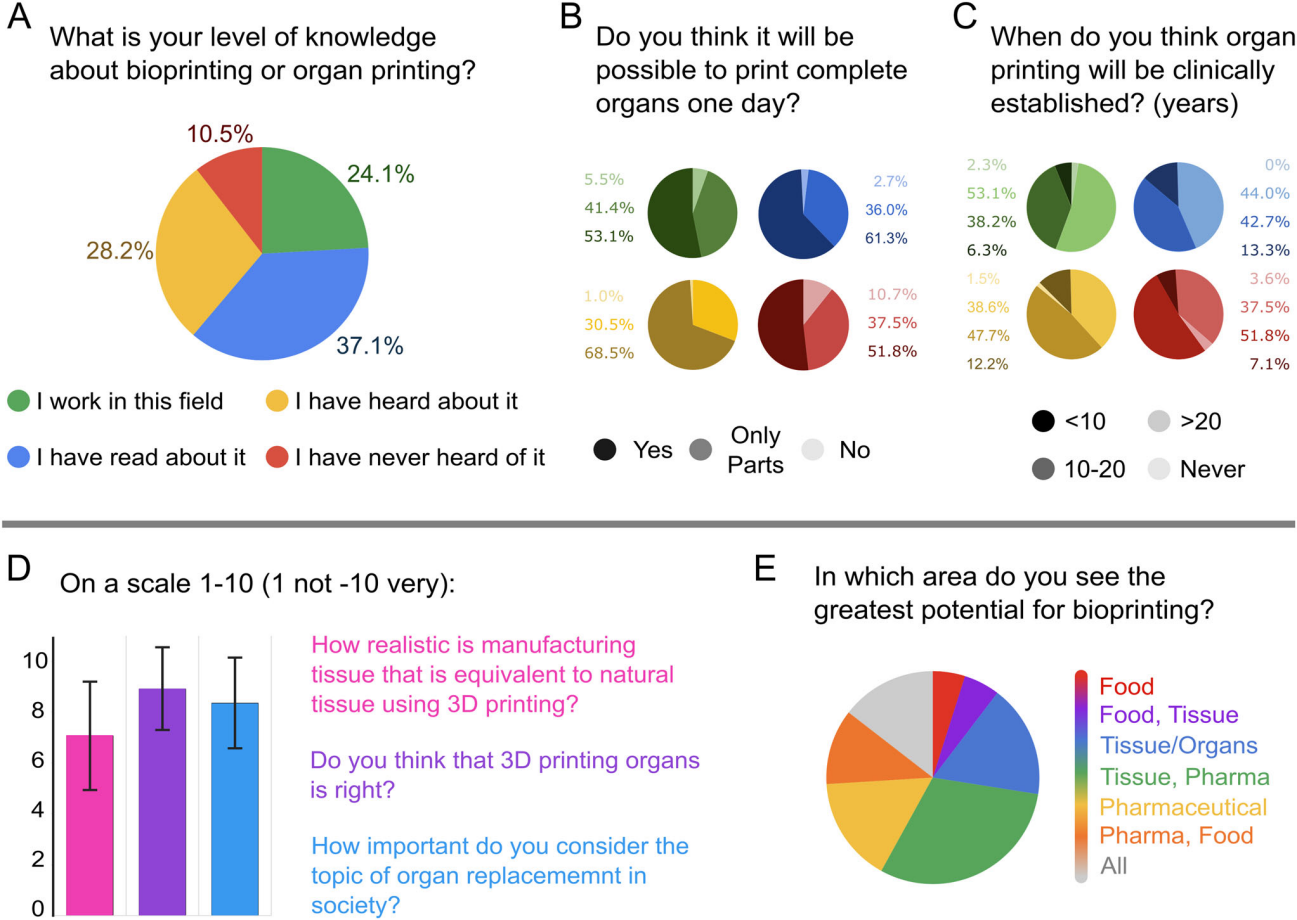
Results from the survey conducted on the feasibility of printing organs in the near future. A) What is your level of knowledge about bioprinting or organ printing? That question allows us to divide the population into four categories depending on the level of expertise: I work in this field, I have read about it, I have heard about it, and I have never heard of it. B) Do you think it will be possible to print complete organs one day? Each pie chart represents a different level of expertise. C) When do you think organ printing will be clinically established? Each pie chart represents a different level of expertise. D) Shows the importance of the topic on a scale from 1–10 regardless of the level of expertise, where “1” corresponds to “I don't agree/Negative”, whereas “10” corresponds to “I very much agree/Positive”. Pink bar plot: How realistic is manufacturing tissue that is equivalent to native tissue using 3D printing? Purple bar plot: Do you think that 3D printing organs is right? Blue bar plot: How important do you consider the topic of organ replacement in society? E) In which areas do you see the greatest potential for bioprinting? The results are depicted via pie charts. Total number of participants: 531.

To better understand the expectations and current awareness of biofabrication in the population, including its potential societal implications, a survey was conducted as part of this overview (**Figure** **2**; Supporting Information S1). This survey gathered insights from both experts in the field and the general public, with a focus on the anticipated applications of biofabrication in healthcare, pharmaceutical testing, tissue and organ replacement, and even food production. The results from this survey highlight broader expectations for biofabrication, illustrating both its promise and its challenges. The survey was conducted among 531 people with varying levels of knowledge on the topic, from the biofabricators themselves to the laymen themselves, and all had opinions on bioprinting organs. The results were split according to the self‐proclaimed level of expertise of the participants (Figure 2A; Figure S1, Supporting Information).

The survey revealed that the experts involved in the field have a conservative perspective (Figure 2B, C). This is most likely because the experts are directly involved in developing these approaches and techniques and in solving problems on a daily basis. In addition to the experts, the most skeptical were people who never thought about bioprinting organs. However, those with “some form of knowledge” about bioprinting were the most hopeful and optimistic about the outcomes of this emerging scientific field. When confronted with trying to pinpoint a certain realistic time projection for organ printing (Figure 2C), the participants’ opinions diverged. On the one hand, the majority of experts tended to estimate 20 or more years, whereas the other groups believed that shorter time periods would be realistically achievable.

Overall, the interviewed public thinks that the topic of organ replacement and production of artificial organs is highly relevant for society, scoring an average of 8.3 on a scale of 1–10, with very close results among all groups (Figure 2D); when asked if the production of organs by bioprinting is “right” to do, the average answer also scored 8.9 out of 10 points, with some more reservations from the nonknowledgeable cohort (Figure 2D). The general idea of the realism of ever achieving the production of bioprinted organs is above average (7.0 out of 10), with very encouraging results from nonexperts (Figure 2D). Overall, the topic is widely supported across society, and it is expected that advances in the field will play a crucial role in the future development of functional organ printing.

Despite the accuracy of approximating the time needed for printing organs established in clinical practice, most survey participants believe that it will be achievable to at least produce tissues or parts of organs (Figure 2E). Interestingly, the audience agrees that bioprinted tissues could also be used in the pharmaceutical industry and even in the production of “food of the future,” such as cellular‐agricultured meat. The major issues highlighted in the survey were as follows: finding suitable materials and the challenge of keeping multiple cell types alive enough for proper maturation.

In this overview article, written from students to students with various scientific backgrounds, the current state of the technology in the context of materials, processes, and interdisciplinary strategies for the development and production of functional tissues is examined. On the basis of the most promising studies, it provides an assessment of the scientific field of bioprinting and its current status and concludes that progress still needs to be made closer to the goal of organ printing.

## 2 Biofabrication Aspects in Brief

**Figure 3:**
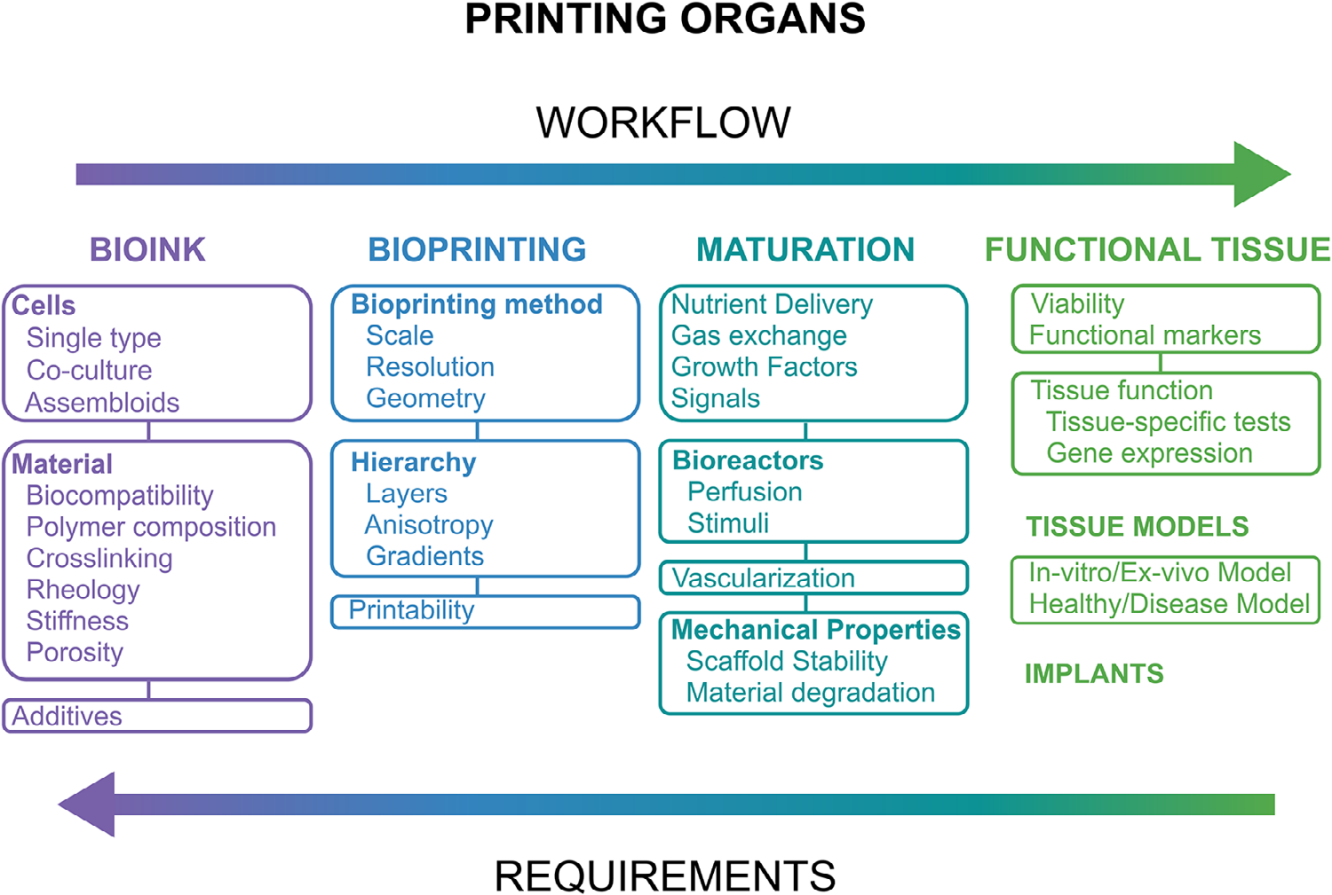
Workflow for organ bioprinting: from material design to printing, maturation, and testing of tissue functionality. Left to right: bioink, bioprinting, maturation, and functional tissue/tissue models. The bioink shows subchapters with examples: cells, materials, and additives. The bioprinting subchapters represent bioprinting methods, hierarchies, and printability. Maturation emphasizes nutrient delivery, gas exchange, growth factors, signals, bioreactors, vascularization, and mechanical properties. Functional tissue shows viability, tissue function, and further tissue models. Reading from right to left: The tissue dictates the requirements for choosing a certain maturation process, bioprinting technique, and material.

The field of biofabrication was originally defined as “*the production of complex living and nonliving biological products from raw materials such as living cells, molecules, extracellular matrices, and biomaterials*”^[^ ^24^ ^]^ and more specifically as “*the automated generation of biologically functional products with structural organization from living cells, bioactive molecules, biomaterials, cell aggregates such as microtissues, or hybrid cell–material constructs, through bioprinting or bioassembly and subsequent tissue maturation processes*”.^[^ ^21^, ^24^ ^]^ While biofabrication has tremendous potential, significant advancements in processes, materials, and technology are still needed to make clinical application successful. Scalability and costs remain critical challenges for biofabrication, and overcoming them lies in interdisciplinary collaboration. Engineers, life scientists, and industry stakeholders must work together to refine biofabrication techniques, define translatable goals, and establish validation protocols. Collaboration must flow in both directions: from materials science and bioink manufacturing to the engineering of bioprinters and the biological development of artificial organs, reflecting the natural workflow of tissue formation. Conversely, the specific requirements of each organ should also guide advancements in biofabrication technologies, ensuring that engineering approaches are tailored to the complexities of human physiology (**Figure** **3**). Future progress will depend on interdisciplinary collaboration, continuous innovation, and public acceptance, all of which are essential for the success of this transformative technology.

### 2.1 Bioinks

**Figure 4:**
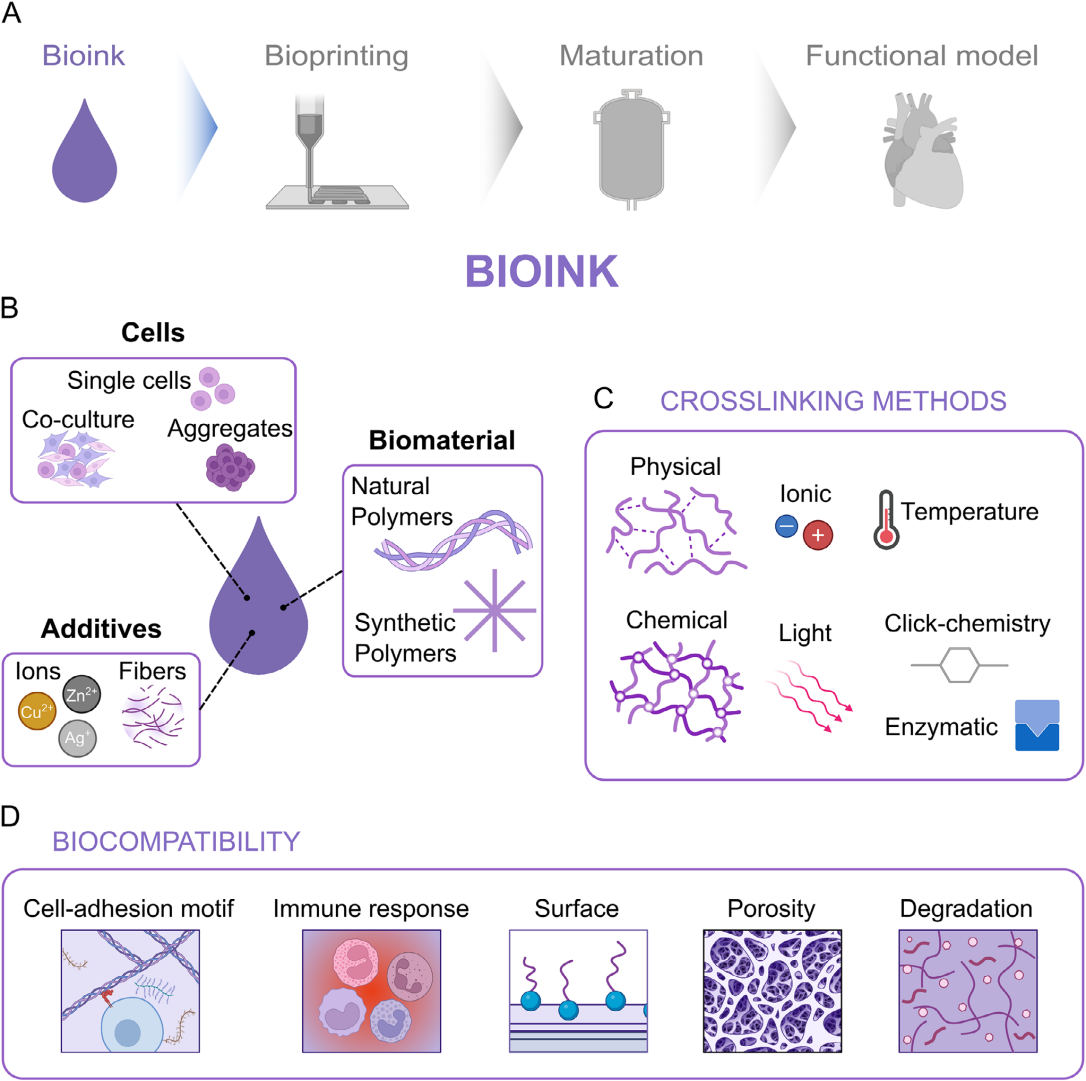
A) Biofabrication workflow: bioink. B) Formulation of bioinks: cells represent the main component, and they are eventually embedded within a natural or synthetic hydrogel matrix. These formulations are often enhanced with various additives to optimize hydrogel functionality. C) Crosslinking methods play a crucial role in stabilizing printed structures. D) Biocompatibility considerations, such as physical properties (cell‐adhesion motif, surface, porosity, degradation) and host response, are essential for developing implantable materials. Created in BioRender. Mussoni, C. (2025) https://BioRender.com/yid0ai3.

In the context of bioprinting organs, the first crucial step is to decide which type of material will host the cells and be printed (**Figure** **4**A). Materials exploited in contact with living tissues, organisms, or microorganisms^[^ ^25^ ^]^ are defined as biomaterials. When these biomaterials are formulated with living cells for use in automated biofabrication, they are termed bioinks, defined as “*a formulation of cells that is suitable to be processed by an automated biofabrication technology*,”^[^ ^26^ ^]^ where cells are a mandatory component, whereas cell‐free formulations are termed biomaterial inks.^[^ ^21^ ^]^ Biomaterials include metals, ceramics, polymers, and composites, all of which are extensively used in medicine for applications such as orthopedics, dental implants, and cardiovascular devices.^[^ ^27^ ^]^ However, for the purpose of this review, the focus lies exclusively on polymers that can be used in a bioink or in biomaterial inks.

In bioprinting, hydrophilic polymers are used to encapsulate cells, which can be prepared using a single cell type to study specific cell behaviors, cocultures to investigate interactions between different cell types, or aggregates to closely mimic native tissue structures. These formulations can eventually be supplemented with additives that improve the stability of the construct or functionally support the cells (Figure 4B). Hydrophilic polymers can undergo a crosslinking process to form hydrogels, which are networks of polymer chains that are highly absorbent and can retain large amounts of water relative to their dry weight, forming a gel‐like substance. The hydrophilic functional groups attached to the polymeric backbone enable hydrogels to absorb water, while the cross‐links between network chains provide resistance to dissolution. The ability of hydrogels to absorb water derives from hydrophilic functional groups attached to the polymeric backbone, whereas their resistance to dissolution arises from cross‐links between network chains. These features are essential for providing functional support mimicking the extracellular matrix (ECM), ensuring smooth extrusion through bioprinter nozzles, and maintaining the structural integrity of printed constructs.^[^ ^28^ ^]^

To bioprint functional tissue, it is fundamental to resemble or mimic the ECM. The ECM provides not only the environment for cells but also required biochemical cues. It is a sophisticated network composed of more than 300 proteins,^[^ ^29^ ^]^ providing growth factors and bioactive molecules.^[^ ^30^ ^]^ The components of the ECM link together to form a structurally stable composite, contributing to the mechanical properties of tissues. It is a highly dynamic entity that is vital for determining and controlling the most fundamental behaviors of cells, such as proliferation, adhesion, migration, polarity, differentiation, and apoptosis.^[^ ^31^, ^32^ ^]^ Therefore, it is not surprising that each organ has its own specialized ECM, with varying properties and precise protein compositions. The major components are collagens, proteoglycans, elastin, and cell‐binding glycoproteins, each with distinct physical and biochemical properties. Cells form contact with their specific ECM through a variety of surface proteins that recognize and bind distinct ECM components. The most important of these are integrins, which connect to proteins such as fibronectin, collagen, laminin, and vitronectin, linking the ECM to the actin cytoskeleton and mediating crucial signaling. Integrins enable cells to transmit signals and connections between intra‐ and extracellular compartments, highlighting the indispensability of the ECM for living tissue.^[^ ^33^ ^]^ Other receptors include discoidin domain receptors that bind collagen and help regulate matrix remodeling, dystroglycans that anchor cells to laminin‐rich basement membranes, syndecans that engage ECM proteins via their heparan sulfate chains, and CD44, which primarily interacts with hyaluronan. Together, these proteins enable cells to sense, adhere to, and respond dynamically to their surrounding matrix, thereby guiding processes such as migration, differentiation, and tissue organization.

#### 2.1.1 Polymers

Biomaterials can be categorized on the basis of their origin into natural, synthetic, or composite types. The origin is critical, as it directly affects the ability of a biomaterial to support cell viability and function, as well as its printability and structural integrity. Natural bioinks are well known to offer excellent biological compatibility and ECM‐mimicking properties, which are essential for creating functional tissue constructs, although their printability can be variable and challenging to control. Synthetic bioinks provide greater tunability and consistency during the printing process, but they may require additional modifications to achieve the same level of biological performance as natural bioinks. Additionally, composite bioinks, which combine natural and synthetic components, are emerging as versatile options. These composites can offer the biological cues and biocompatibility of natural materials alongside the mechanical strength and tunable properties of synthetic materials. The materials used for bioprinting must fulfill several requirements to be suitable as bioinks; therefore, the choice between natural, synthetic, or composite bioinks depends on the specific requirements of the bioprinting application, balancing the need for biological functionality with the practical aspects of the printing process. Moreover, certain polymers can be triggered to change shape by a stimulus, such as a change in pH or temperature. The combination of polymers with such different characteristics or triggers has led to the exploitation of these changes to build dynamic structures that form and unform over time, therefore introducing the concept of a 4th dimension, leading to the term 4D bioprinting.^[^ ^34^, ^35^ ^]^ The biomaterials described below represent only a fraction of the wide array of natural and synthetic materials with potential for biofabrication applications.^[^ ^36^, ^37^ ^]^ These examples were selected on the basis of their intrinsic properties, which align well with the specific requirements of the research outlined in later sections.

##### Natural Polymers

Nature already provides the perfect environment for cells through the ECM, making it a logical choice for biomaterial development. Therefore, different studies have utilized tissue‐specific decellularized ECM (dECM) to support cell culture. dECM is obtained by removing cellular components from tissues while preserving the structural and biochemical integrity of the native ECM. This process retains essential bioactive molecules such as collagen, proteoglycans, and growth factors, enabling the dECM to serve as a multifunctional scaffold. Its ability to support cell attachment, proliferation, migration, differentiation, and maturation is largely attributed to its preserved cell signaling properties. For example, Matrigel, a commercially available basement‐membrane‐like matrix, is the most commonly used natural hydrogel for encapsulating neuronal cells.^[^ ^38^ ^]^ However, it has certain drawbacks: it is tumor‐derived, exhibits batch‐to‐batch variability, contains tumor‐derived proteins, and lacks stability in long‐term cultures.^[^ ^39^ ^]^

Another widely adopted approach to replicate the ECM is the use of natural polymers, which are classified as either proteins or polysaccharides. These natural polymers can either be part of the ECM or can be naturally occurring polymers, which are needed to mimic the properties of ECM molecules for bioprinting. Collagen, for example, represents a molecule that naturally occurs within the ECM. Gelatin, on the other hand, is a natural protein that is not present within the ECM but can be used to mimic its properties. Many of these materials have the following key properties: biocompatibility, cell binding sites, potential to form ECM‐like microstructures, and the ability to be degraded by embedded cells^[^ ^40^ ^]^ (**Table** **1**).

**Table 1:** Examples of studies of different crosslinking and printing techniques for bioinks.

Biomaterial	Crosslinking	Printing technique	Ref.
Natural polymers			
Collagen	pH‐mediated Thermal induction	Extrusion based Droplet based	[143]
Thiolated Hyaluronic acid	pH‐mediated Michael addition Photopolymerization	Extrusion based	[144]
Methacrylated Hyaluronic acid	Photopolymerization	Extrusion based	[144]
Gelatin	Thermal induction	Extrusion based Laser based	[145]
Methacrylated Gelatin	Photopolymerization	Extrusion based Droplet based In situ Light based	[146]
Fibrinogen	Enzymatic reaction	Droplet based	[147]
Chitosan	pH mediated	Extrusion based	[148]
Silk fibroin	Ultrasonic induction	Extrusion based Light based	[149]
Alginate	Ionic	Extrusion based Light based Droplet based	[150]
Gellan gum	Ionic	Extrusion based	[151]
Xanthan gum	Ionic	Extrusion based	[152]
Polyethylene glycol	Thermal induction	Extrusion based	[153]
Acrylated polyethylene glycol or multiarm polyethylene glycol	Photopolymerization	Extrusion based Light based	[153]
Pluronic F127	Thermal induction	Extrusion based	[153]
Polycaprolacton	Melt by high temperature	Melt electrowriting	[98]

##### Proteins

Collagens are the most abundant proteins in mammals and are found in the ECM of various tissues, including skin, bone, ligament, tendon, and cartilage.^[^ ^41^ ^]^ Among the 28 distinct types of collagens within the collagen superfamily,^[^ ^42^ ^]^ type I collagen is crucial in our bodies and forms a significant portion of the matrix materials used in biofabrication. All collagens are characterized by a highly repetitive tripeptide sequence that has glycine as every third amino acid. Therefore, the sequence is commonly depicted as (Gly‐Xaa‐Yaa)n, where the Xaa and Yaa positions are occupied by a disproportionate fraction of the amino acid proline and its hydroxylated form hydroxyproline, which, together with hydroxylysine, play a pivotal role in collagen stability^[^ ^43^ ^]^ in mammals. This composition allows three polypeptide chains, called α‐chains, to tightly pack a homo‐ or heterotrimeric right‐handed superhelix. Collagens are classified into fibrillar and nonfibrillar collagens on the basis of their ability to hierarchically arrange into supramolecular fibrils. Fibrillar collagens, including types I, II, III, V, XI, XXIV, and XXVII, play structural roles or assist in the fibrillogenesis of other collagens.^[^ ^44^ ^]^ The widespread availability of collagen is an advantage, but batch‐to‐batch variation and the potential risk of disease transmission are notable disadvantages. Advances in the production of collagen‐like proteins and recombinant collagen, which are stabilized through charged amino acid pairs, offer promising alternatives.^[^ ^45^ ^]^

Gelatin, a product of the thermal denaturation of collagen, presents an appealing alternative in bioprinting to collagen because of its similar properties but lower cost and easier production.^[^ ^46^ ^]^ Gelatin varies in molecular weight and isoelectric point depending on the source of the collagen and the manufacturing process. It is derived mainly from sources rich in collagen I, with the amino acid composition varying between species. Chemical modifications of gelatin can enhance its bioprinting properties. One notable modification is gelatin methacrylate (GelMA), which combines the benefits of gelatin with additional functional groups that allow for photopolymerization. This modification enhances the mechanical properties and stability of gelatin, making GelMA a popular choice in bioprinting applications. Other modifications of gelatin used in biofabrication include thiol‐ene clickable gelatine (GelAGE),^[^ ^47^ ^]^ which is suitable for multiplatform fabrication, and gelatin‐norbornene (Gel‐NOR).^[^ ^48^ ^]^

Another alternative to collagen is represented by fibrinogen, which can be easily obtained from patients’ blood. Indeed, fibrinogen is the inactive form of fibrin, a protein involved in the natural repair process of tissues and in the coagulation cascade. When combined with the protease thrombin, fibrinogen is cleaved and converted to fibrin, which self‐assembles into a hydrogel. Since it is similar to what cells would otherwise naturally encounter in a wound, it can be used as an autologous source for a scaffold but has the advantage of reducing the risk of immunological incompatibility.^[^ ^49^ ^]^ Fibrin hydrogels are often used for applications such as generating or repairing nerve^[^ ^50^ ^]^ and cardiac tissues.^[^ ^51^ ^]^ It is usually casted but can be bioprinted with additives. Thrombin is added after deposition to crosslink the hydrogel.^[^ ^52^ ^]^

Among protein‐based materials, silk has gained considerable attention because of its biocompatibility and low immunogenicity.^[^ ^53^ ^]^ Silk is a protein‐based fiber‐forming material produced by living organisms,^[^ ^54^ ^]^ such as silkworms and nearly all spider species. Silk fibroin is characterized by highly repetitive domains arranged in a coblock‐polymer‐like fashion, flanked by globular domains at the N‐ and C‐terminus, which play a role in solubility and storage.^[^ ^55^ ^]^ The eADF4(C16) protein is a recombinant spider silk protein used in many bioprinting approaches,^[^ ^56^, ^57^ ^]^ as it can be used to produce physically cross‐linked hydrogels^[^ ^58^ ^]^ processable by direct extrusion printing.^[^ ^59^, ^60^ ^]^ Recombinant silk further allows tailoring of the amino acid sequence through genetic engineering to alter the overall net charge of the protein,^[^ ^61^ ^]^ which was shown to have a positive effect on the adhesion of human‐induced pluripotent stem cell (hiPSC)‐derived cardiomyocytes or to contain cell‐binding motifs for cell‐selective adhesion.^[^ ^62^ ^]^ The RGD‐tagged variant was found to support de novo tissue formation in vivo^[^ ^63^ ^]^ and, furthermore, has been utilized to stabilize collagenI hydrogels via an in‐gel printing approach whereby the collagen was extruded into a bath containing eADF4(C16) and the silk content subsequently precipitated by using a phosphate buffer, interconnecting the collagen fibers and yielding a more stable composite material.^[^ ^64^ ^]^ By introducing methacrylate groups into silk fibroin, it is possible to improve the mechanical properties and biocompatibility. Methacrylated silk retains the high tensile strength and elasticity of natural silk, combined with the ability to undergo photocrosslinking.

Elastin‐like proteins/polypeptides (ELPs) are also recombinant proteins that mimic the properties of natural elastin, which is a key structural protein in the ECM that endows it with elastic properties.^[^ ^65^ ^]^ Natural elastin is composed of cross‐linked tropoelastin monomers that are rich in hydrophobic amino acids. ELPs mimic the repetitive amino acid sequence found in elastin, typically VPGXG, with X being any other amino acid except proline.^[^ ^66^ ^]^ ELPs are soluble in water below a certain temperature and become insoluble and self‐assemble above it.^[^ ^67^ ^]^ These materials are attractive to cells and thus facilitate good cell proliferation; hence, they are good hydrogel matrix components. They can also be modified to crosslink via different mechanisms in addition to self‐assembly and processed into materials with viscoelastic mechanical behaviors similar to those of living tissue.^[^ ^68^ ^]^

##### Polysaccharides

Hyaluronic acid (HA) is a naturally occurring biopolymer found in the ECM of connective tissues and is known for its high biocompatibility, viscoelasticity, and exceptional water‐retention ability. HA, which is composed of repeating disaccharide units of N‐acetylglucosamine and glucuronic acid, plays a crucial role in tissue hydration, elasticity, and mechanical integrity.^[^ ^69^ ^]^ In bioprinting, HA is used to create bioinks that closely mimic the natural ECM environment, promoting cell viability, adhesion, migration, and proliferation. Customizing HA‐based bioinks involves modifying the molecular weight and concentration to achieve optimal rheological properties, such as increased viscosity for better printability and structural stability.^[^ ^70^ ^]^ Modifying HA with agents such as methacrylate enhances its mechanical properties and provides tunable degradation rates, making HA‐based bioinks suitable for a wide range of tissue engineering applications, including those involving cartilage, skin, and vascular tissues.^[^ ^71^ ^]^

Derived from brown seaweed or bacteria, alginate is a nonbranching, anionic polysaccharide composed of guluronic and mannuronic acid units, making its structure similar to the hyaluronic acid of the ECM.^[^ ^72^ ^]^ Alginate can be crosslinked by divalent cations such as Ca^2+^, a gentle process that enhances its suitability for bioinks.^[^ ^73^, ^74^ ^]^ However, pristine alginate has poor degradability and lacks cell adhesion motifs. To address these drawbacks, alginate is commonly used in its oxidized form, known as alginate‐di‐aldehyde (ADA), and combines with components that provide cell adhesion sequences, such as RGD sequences.^[^ ^75^ ^]^ Several in‐depth studies have been conducted on alginate and ADA‐based bioinks to assess their material properties and biological potential. For example, various alginates can be characterized, and ADAs can be produced to determine the most suitable molecular weight, degree of oxidation, and polymer concentration for tissue engineering.^[^ ^76^ ^]^

Chitosan is a linear polysaccharide obtained from partially deacetylated chitin, the primary structure of the exoskeleton of crustaceans and insects, as well as in some algae and the cell wall of fungi.^[^ ^77^ ^]^ Structurally, chitosan has many primary amino groups along its backbone, and its overall structure is somewhat reminiscent of the glycosaminoglycans contained in the ECM.^[^ ^78^ ^]^ Chitosan is usually soluble only at an acidic pH and physically crosslinks to form a hydrogel when the pH is increased or when chemical crosslinking of chitosan occurs. Additives such as glycerol phosphate may be processed at neutral pH values and form a thermosensitive system that gels at approximately 37 °C. Nonetheless, owing to the relatively harsh processing conditions for chitosan, it is not often used in conjunction with cells before the crosslinking process is finished.^[^ ^79^ ^]^

##### Synthetic Polymers

Natural materials have many advantageous properties for biofabrication, such as the presence of adhesion motifs, inherent biocompatibility, and often high availability. However, they also have several drawbacks, including batch‐to‐batch variability, complex purification processes, immune reactions, and the risk of pathogen transmission. Many of these limitations can be mitigated by selecting synthetic materials as alternatives.^[^ ^80^ ^]^ Their mechanical properties can be fine‐tuned by adjusting factors such as the polymer concentration, crosslinking, and molecular weight, allowing for the fabrication of complex three‐dimensional scaffolds. Additionally, synthetic materials can be customized with integrin‐binding motifs, growth factors, or other biological stimuli during production.^[^ ^81^ ^]^

Polyethylene glycol (PEG) has received considerable research attention as a synthetic material because of its extensive chemical versatility.^[^ ^82^ ^]^ Indeed, PEG can be tuned by converting hydroxyl groups into various functional groups. These modifications enable crosslinking via UV light by attaching acrylic or methacrylic groups, forming PEGDA or PEGDMA.^[^ ^82^ ^]^ However, PEG‐based hydrogels initially lacked the capacity for biological interaction with embedded cells. Several strategies exist to address this limitation. For example, it can be combined with a natural hydrogel to improve bioactivity^[^ ^83^ ^]^ or enhance cell adhesion by conjugating PEG with adhesive peptides derived from laminin and fibronectin. In addition, the use of multiarm PEG instead of linear PEG increases the potential number of easily accessible binding sites along the PEG chain. These can then be used to crosslink with more of the hydrogel‐forming polymers at once, resulting in a more interconnected network.^[^ ^84^ ^]^ Alternatively, if mixed at the appropriate ratio, they can be used to attach/immobilize bioactive molecules, such as laminin or growth factors, within the hydrogel to make it more attractive.^[^ ^85^ ^]^ With drop‐on‐demand printing, the creation of cell‐laden hydrogel particles that facilitated high cell‒material interactions, with embedded cells displaying favorable morphology is possible.^[^ ^86^ ^]^ These results highlight the versatility of PEG as a biomaterial and its potential for high‐precision 3D fabrication methods.^[^ ^87^ ^]^

Polyoxazolines, especially poly(2‐oxazoline) (POx), have also gained recent research attention. POx offers diverse network formation methods^[^ ^88^ ^]^ and shows excellent cytocompatibility, regardless of the crosslinking method applied.^[^ ^89^, ^90^ ^]^ Like PEG, POx can be conjugated with cell adhesion motifs, and its chemical structure facilitates this modification.^[^ ^91^ ^]^ Fibrinogen‐conjugated POx hydrogels were developed, providing a conducive environment for chondrocyte growth and cartilage development. Additionally, the hydrogel demonstrated the ability to crosslink with surrounding tissue, making it a promising candidate for injectable treatments for chondral defects.^[^ ^92^ ^]^ This biomaterial has also been modified to develop an ink based on two types of POx copolymer units, which exhibit rapid thermoresponsive inverse gelation. This property makes it particularly suitable for 3D printing. Rheological assessments confirmed the shear‐thinning behavior of the bioink, and cytotoxicity testing indicated excellent suitability for bioprinting.^[^ ^93^ ^]^

Owing to its thermoresponsive properties, Pluronic 127, also known as Poloxamer 407, is widely utilized as a sacrificial ink in biofabrication.^[^ ^94^, ^95^ ^]^ At low temperatures, it remains in a liquid state, while it solidifies into a gel at physiological temperatures. While liquid or semiliquid, it allows precise extrusion and support of the bioink used during 3D bioprinting. Once deposited, it solidifies into a gel at physiological temperatures, forming temporary structures within the printed scaffold.^[^ ^96^, ^97^ ^]^ These structures can be easily removed by lowering the temperature or using a solvent, leaving behind the intricate channels or voids necessary for creating complex tissue constructs and enhancing nutrient diffusion and cell viability in tissue engineering applications.

Other hydrophobic polymers, such as polycaprolactone (PCL) and poly lactic acid (PLA), are suitable as biomaterial inks. Their mechanical properties, including flexibility and strength, make them excellent materials for creating scaffolds that can support cell growth and tissue development.^[^ ^98^ ^]^ Biofabrication methods are often used to produce reinforcements for bone and cartilage because of their slow degradation rate, which provides prolonged structural support during tissue regeneration.^[^ ^99^ ^]^

#### 2.1.2 Additives

Bioinks can also be mixed with solid additives (Figure 4B) to improve printability and stability, facilitate drug release, or direct cell migration and differentiation. Ideally, these materials degrade over time, allowing tissue maturation after fulfilling their initial purpose.

##### Inorganic Particles

One type of filler material for bioinks includes inorganic particles. When blended into the ink, these particles enhance rheological properties and print stability while also providing surfaces for cells to attach to and modulate their behavior. Inorganic particle fillers, with materials such as hydroxyapatite, β‐TCP, and bioactive glasses, are commonly used in bone tissue‐like constructs. The inclusion of biologically active ions such as Zn^2^⁺, Sr^2^⁺, Ag⁺, Cu^2^⁺, and drugs can further tailor the material to the specific needs of the tissue.^[^ ^100^ ^]^ For example, blending an alginate dialdehyde/gelatin bioink with Cu‐doped mesoporous bioactive glass nanoparticles improved the rheological properties, structural stability, and shape fidelity of printed constructs. This ink, when used to print human osteosarcoma cells and immortalized mouse bone marrow‐derived stem cells, supported high cell survival. It also induced osteogenic differentiation and angiogenesis of primary mouse bone marrow stromal cells without additional growth factors, likely due to ion stimulation.^[^ ^101^ ^]^ In addition to inorganic components, a blend of organic and inorganic materials, as observed in natural bone, can create a more native signaling environment for cells.^[^ ^102^ ^]^

##### Short Polymer Fibers

Another type of filler material are cut polymer fibers. Fibers of various natural and synthetic polymers are commonly used to reinforce hydrogels, offering enhanced surfaces for cell guidance across biofabrication applications. To incorporate such fibers into inks, they must be shortened to appropriate sizes to avoid clogging the bioprinter nozzle and ensure even distribution without aggregation. This can be achieved by fragmentation^[^ ^57^, ^103^ ^]^ or by cutting them to the desired lengths.^[^ ^104^ ^]^ Once incorporated, these fibrous fillers alter the rheological behavior of the ink, improve the mechanical stability and resolution of printed constructs, and provide a mechanism for cell alignment. Fibers can also be functionalized with different factors or used as carriers for controlled drug release. For example, adding cellulose nanofibers or small PCL fragments modified the rheological properties of alginate and Pluronic acid, affecting the viscosity of the complex and strut spreading after deposition.^[^ ^105^ ^]^ It has also been demonstrated that shape fidelity in alginate/gelatin inks was improved by adding gelatin fibers, although no cells were printed with this ink.^[^ ^106^ ^]^ Similarly, adding nanocellulose fibers improved the printability and shape fidelity of alginate bioinks, which could then be printed with melanoma cells and spheroids, offering better cellular interaction with the hydrogel matrix than pure alginate.^[^ ^107^ ^]^ Incorporating PCL fragments into cell‐inert recombinant spider silk bioinks also improved cell proliferation after printing, as the fragments provided a more favorable attachment surface than the base ink alone.^[^ ^108^ ^]^ If fibers are sufficiently long, they can align during printing because of shear forces and direct flow during deposition. This induced fiber alignment can guide cells and promote alignment, resulting in tissue anisotropy.^[^ ^109^ ^]^

### 2.2 Crosslinking Mechanisms

Crosslinking is a process needed for the stabilization of bioinks. For extrusion‐based printing, crosslinking is required for bioinks to be printable as well as for the long‐term stability of the printed construct. The type of crosslinking can affect the network density, bioink long‐term stability, and mechanical properties, e.g., stress relaxation behavior^[^ ^110^, ^111^ ^]^ and Young's modulus; therefore, choosing the right crosslinking mechanism can influence the final construct. In some cases, one crosslinking strategy is sufficient to form a stable hydrogel, but the mechanical properties are not adequate to be printable and eventually be suitable as a bioink. Therefore, combining multiple crosslinking strategies is common. In general, we can distinguish between two different crosslinking strategies: physical crosslinking and chemical crosslinking (Figure 4C). Both strategies are commonly used for extrusion‐based bioink gelation, i.e., the transition between a fluid and a viscous bioink.

#### 2.2.1 Physical Crosslinking

The gelation of hydrogels or bioinks that undergo physical crosslinking occurs through noncovalent bonds. Owing to the absence of covalent bonds, bioinks resulting from physical crosslinking often have different mechanical responses, as the polymer network can be reorganized under stress, leading to faster stress relaxation.^[^ ^111^, ^112^ ^]^ Although mechanically weaker than chemically crosslinked bioinks, these bioinks demonstrate high biocompatibility and low cytotoxicity due to the absence of chemical crosslinking agents. They have been successfully tested across various 3D printing techniques and with a wide range of cell types.^[^ ^113^, ^114^ ^]^

#### 2.2.2 Ionic and Electrostatic Interactions

Crosslinking based o ionic or electrostatic interactions relies on the attraction between oppositely charged molecules. In ionic interactions, multivalent cations (e.g., CaCl_2_) are added as crosslinking agents to induce gelation, while electrostatic interactions leverage ionic groups already present within the polymer structure. Both techniques can be performed at room temperature and are not light sensitive, making them convenient and accessible options for bioinks. A prominent example in biofabrication are alginate‐based bioinks, where alginate crosslinks via the carboxylic groups of neighboring polymer chains in the presence of multivalent cations, resulting in gelation.^[^ ^114^, ^115^ ^]^ The stiffness of the final bioink, as well as the viability and proliferation of embedded cells, can vary depending on the alginate's molecular weight, crosslinking duration, and chosen crosslinker.^[^ ^116^, ^117^ ^]^ This cross‐linking approach, known as metal coordination, is also applicable to other bioinks, such as gellan gum, a negatively charged polysaccharide that cross‐links in the presence of cations. However, gellan gum‐based bioinks are generally not mechanically stable enough on their own, necessitating combination with other polymers and additional crosslinking.^[^ ^118^, ^119^ ^]^ Hydrogels purely reliant on electrostatic interactions are often too weak for printing, so polymers such as chitosan, which are suitable for electrostatic crosslinking, are typically combined with other polymers or crosslinking methods to increase their usability as bioinks.^[^ ^120^, ^121^ ^]^

#### 2.2.3 Hydrophobic Interactions

Crosslinking can also be achieved through hydrophobic interactions in water‐soluble polymers that have hydrophobic groups or chains. These interactions can be induced either by temperature changes or ultrasonic treatment. Temperature‐sensitive hydrogels differ on the basis of their response to either the lower critical solution temperature (LCST) or the upper critical solution temperature (UCST). Polymers responsive to the LCST remain soluble below the LCST but become hydrophobic above it, leading to micelle formation or coil‒helix transitions, which induce gelation. In contrast, UCST‐responsive polymers form hydrogels below their UCST.^[^ ^122^, ^123^ ^]^ Gelatin, derived from hydrolyzed collagen, is the most widely used thermosensitive polymer in biofabrication. At lower temperatures, gelatin forms triple helices from single‐coil structures through hydrogen bonding and van der Waals forces.^[^ ^122^ ^]^ However, despite the high biocompatibility of gelatin and collagen, unmodified hydrogels of these materials are not printable and often require chemical modifications or additional components to be suitable for printing applications.^[^ ^124^ ^]^ A less common approach for crosslinking bioinks via hydrophobic interactions is sonification. Ultrasonication can induce β‐sheet formation in polymers such as silk fibroin, resulting in physical crosslinking. Like other physical crosslinking methods, sonification is frequently used in combination with other crosslinking mechanisms.^[^ ^125^, ^126^ ^]^

#### 2.2.4 Hydrogen Bonding, Crystallization, and Host–Guest Interactions

Other common physical crosslinking methods, such as hydrogen bonding, crystallization, and host–guest interactions, can be used to form hydrogels. Hydrogels produced solely through these methods are often too weak and lack the stability required for printing.^[^ ^113^, ^114^, ^127^ ^]^ However, hydrogen bonding and guest–host interactions can be deliberately engineered to enhance printability.^[^ ^128^ ^]^ These dynamic, reversible bonds act as physical cross‐links that provide shear‐thinning behavior during extrusion and rapid self‐recovery after deposition, enabling smooth flow while maintaining shape fidelity.^[^ ^129^ ^]^

#### 2.2.5 Chemical Crosslinking

While physical crosslinking occurs via noncovalent bonds, chemically crosslinked hydrogels or bioinks are crosslinked via covalent bonds between the polymers. Bioinks, which are crosslinked via chemical crosslinking, often include chemical crosslinkers, e.g., sodium bicarbonate, or photoinitiators, such as Irgacure 2959 or lithium phenyl‐2,4,6‐trimethylbenzoylphosphinate (LAP).^[^ ^113^, ^114^, ^130^ ^]^ Chemically crosslinked bioinks are usually very stable and provide good shape fidelity. Various techniques based on chemical crosslinking, such as photocrosslinking, click chemistry, and enzyme‐catalyzed reactions, have been implemented for bioinks.

#### 2.2.6 Light‐Induced Crosslinking

Photocrosslinking offers the advantage of rapid hydrogel formation under relatively mild conditions, along with relatively straightforward adjustment of the mechanical properties of the bioink. One example of a photosensitive crosslinking mechanism is radical polymerization involving free radicals from photoinitiators reacting with polymer functional groups such as acrylates, resulting in bioink gelation. Alternatively, bioink gelation can be based on thiol groups that bind to unsaturated bonds, such as unsaturated carbon‒carbon bonds. However, this technique can sometimes be limited by the oxidation or formation of disulfide bonds, which may decrease the storage stability of the polymers. Finally, redox‐based reactions involving phenol‐containing polymers can also be applied to bioinks. All three photocrosslinking methods rely on cytocompatible photoinitiators, such as Irgacure 2959, LAP, or riboflavin phosphate. The choice of photocrosslinker also affects the wavelength used for crosslinking. While photocrosslinking is a fast and efficient process for biofabrication, it requires significant optimization of factors such as crosslinking time and light source intensity to minimize the cytotoxicity of the photoinitiator and the wavelength used. Light‐induced crosslinking is very well established in extrusion‐based bioprinting; moreover, it is commonly utilized^[^ ^114^, ^131^ ^]^ in various 3D bioprinting techniques, such as stereolithography and volumetric printing.

#### 2.2.7 Light‐Free Crosslinking

In addition to photosensitive bioinks, many studies have also used chemical crosslinking methods based on light‐free crosslinking approaches. Some prominent examples of chemical reactions that are used for the crosslinking of bioinks are the Diels–Alder reaction, Schiff base formation, and Michael addition. Diels–Alder^[^ ^132^, ^133^ ^]^ is based on a cycloaddition between a diene and an alkene/alkyne. It is a one‐step mechanism without the need for an initiator or catalyst. However, this reaction is not very common for bioinks. More often, Michael addition,^[^ ^134^ ^]^ a reaction between a nucleophile serving as a donor and an electrophilic olefin or alkyne serving as an acceptor, is implemented. The advantages of Michael addition include the large range of possible acceptors as well as the mild conditions. Finally, Schiff base formation^[^ ^86^, ^135^, ^136^ ^]^ can occur between polymers containing alcohol or amine groups and aldehydes, resulting in gelation of the hydrogel. By increasing or decreasing the pH, the reaction time can either increase or decrease. All three types of reactions have been reported to be used in bioinks and enable tunable chemical reactions, allowing adjustment of the crosslinking to specific bioinks and printed structures.^[^ ^137^ ^]^ Bioinks based on click chemistry can also be combined with other crosslinking methods.

#### 2.2.8 Enzyme‐Catalyzed Reaction

In addition to light‐induced and light‐free crosslinking approaches, gelation of bioinks can also be induced via enzymatic reactions. Enzymes operate as catalysts to induce the covalent bonding of natural polymers based on proteins, e.g., collagens, laminin, or silk. For example, transglutaminase, which is commonly used in the food industry,^[^ ^138^ ^]^ can catalyze isopeptide bonds in gelatin‐based bioinks such as GelMA. Another example is the use of tyrosinase,^[^ ^139^ ^]^ an essential enzyme for melanin production, due to its ability to crosslink collagen, which can be used to increase the strength of a collagen‐based bioink.^[^ ^140^ ^]^ In general, enzyme‐catalyzed crosslinking is considered a very gentle and cell‐friendly crosslinking technique; however, many studies combine enzyme‐based crosslinking with other crosslinking techniques to increase the gelation effect and, ultimately, the shape fidelity of the printed construct.^[^ ^141^, ^142^ ^]^

### 2.3 Biocompatibility

Biocompatibility is a fundamental requirement for materials used in tissue engineering and regenerative medicine. It is defined as “*the ability of a material to perform with an appropriate host response in a specific situation*”.^[^ ^154^ ^]^ This definition emphasizes that biocompatibility is not an inherent property of a material but depends significantly on the interaction between the material and the biological environment. The biocompatibility of an implanted material is influenced by both its physical properties—such as surface properties, composition, porosity, sterility, contact duration, and degradation rate—and the host's response to its presence^[^ ^155^ ^]^ (Figure 4D).

#### 2.3.1 Physical Properties

##### Surface Properties

The surface properties of biomaterials—including chemical functionality, hydrophilicity/hydrophobicity, lubricity, smoothness/roughness, and surface energy—play a critical role in determining cell‒material interactions.^[^ ^156^ ^]^ Polymeric biomaterials, which often lack distinct surface properties, can be modified by physical adsorption of molecules or chemical modifications. These modifications can alter surface characteristics, influencing protein adsorption, cell adhesion, and the host response. When biomaterials contact blood, surface energy and wettability become critical factors in controlling thrombosis, as platelets may adhere to and activate on the biomaterial surface. Surface modifications can enhance or reduce protein adhesion, thereby impacting platelet activity and the blood compatibility of the material. For example, high surface roughness can increase bone cell adhesion, which is beneficial for osteoformation, although it may also promote macrophage adhesion, potentially leading to an inflammatory response.^[^ ^157^ ^]^ Surface wettability, which is commonly measured by the contact angle, is particularly significant. Hydrophilic surfaces tend to lower the interfacial free energy, reducing protein adsorption and cell adhesion while improving hemocompatibility. For example, surfaces with contact angles between 70° and 80° are considered optimal for fibroblast growth and proliferation, whereas endothelial cells prefer a contact angle of approximately 39°. Fine‐tuning surface characteristics in this way can significantly enhance the performance and integration of biomaterials in various biological environments.^[^ ^158^ ^]^

##### Porosity

Porosity is a critical attribute of biomaterials used in tissue engineering and refers to the presence of pores within the material and scaffold structure. Pores can exist at different scales: nanoscale (<100 nm) and microscale (<100 µm) within hydrogels, as well as macroscale (100–500 µm) at the scaffold level. Nano‐ and microscopic pores are essential for promoting cell migration, nutrient delivery, and oxygen diffusion throughout the hydrogel,^[^ ^159^ ^]^ especially when the pores are interconnected rather than isolated. Hydrogel porosity also influences the host response; reduced fibrous capsule formation and increased angiogenesis have been observed with subcutaneously implanted scaffolds. Pore interconnectivity and pore size distribution are crucial for cell and tissue migration and growth, whereas total porosity is a vital structural parameter that can negatively impact mechanical properties. However, total porosity alone does not directly correlate with cell or tissue ingrowth, as pore size and interconnectivity play more critical roles.^[^ ^160^ ^]^ High porosity is essential to ensure that newly formed tissues can integrate seamlessly with the scaffold, promoting effective tissue regeneration. However, this must be balanced with the structural integrity of the scaffold. While increased porosity may enhance cellular infiltration, it could also compromise the mechanical strength required to support regenerating tissue. Therefore, an equilibrium between scaffold porosity and the structural solidity needed for the implant is crucial to ensure that the scaffold strength is not compromised.

##### Biodegradability

Biodegradability is a crucial factor in scaffold design for tissue engineering. Depending on their behavior in contact with living tissues, polymeric biomaterials can be categorized as biostable, bioabsorbable (degradable or resorbable), or partially bioabsorbable. During the last two decades of the twentieth century, there was a significant shift from biostable to biodegradable biomaterials for medical and related applications.^[^ ^161^ ^]^ Ideally, a biodegradable scaffold should degrade at a rate that aligns with new tissue formation and maturation, providing temporary support while being gradually replaced by native tissue. This synchronization reduces the need for additional surgeries to remove the scaffold after tissue regeneration is complete, which improves patient outcomes and lowers medical costs. Additionally, the degradation products of biodegradable materials must be nontoxic and easily metabolizable to prevent adverse effects. For example, biodegradable polymers are specifically designed to decompose gradually, making them ideal for applications where long‐term implantation is unnecessary. The biodegradation of polymeric biomaterials involves the cleavage of hydrolytically or enzymatically sensitive bonds within the polymer, resulting in polymer erosion.^[^ ^161^ ^]^

#### 2.3.2 Host Response

The host response, defined as “*the response of a living system to the presence of a material*”, involves various components of the immune system.^[^ ^162^ ^]^ This response can include inflammation, immune activation, or healing processes and is influenced by factors such as the tissue type, microenvironment, and overall health of the host. In this context, ‘biocompatible’ materials are those that do not cause blood clots, induce tumors in surrounding tissues, or provoke immediate immune reactions such as attack, encapsulation, or rejection by the body.^[^ ^163^ ^]^ Historically, biomaterial design focused on minimizing immune reactions by using inert, “passive” materials that would not provoke an adverse immune response. Strategies such as immune‐isolating coatings or hydrogels have been developed to shield implant surfaces from immune cells, thereby reducing protein adhesion and leukocyte activation. However, recent research has revealed that the immune system plays a crucial role in tissue repair and regeneration. The inflammatory response, once seen solely as detrimental, is now recognized as essential for biomaterial integration and effective tissue repair. After biomaterial implantation, the inflammatory response may lead to a foreign body response, which is characterized by fibrous encapsulation and can result in implant failure. However, when properly managed, the inflammatory response can be harnessed to improve biomaterial–tissue integration, promoting angiogenesis and cellular infiltration—processes essential for tissue regeneration.^[^ ^164^ ^]^

### 2.4 Bioprinting

**Figure 5:**
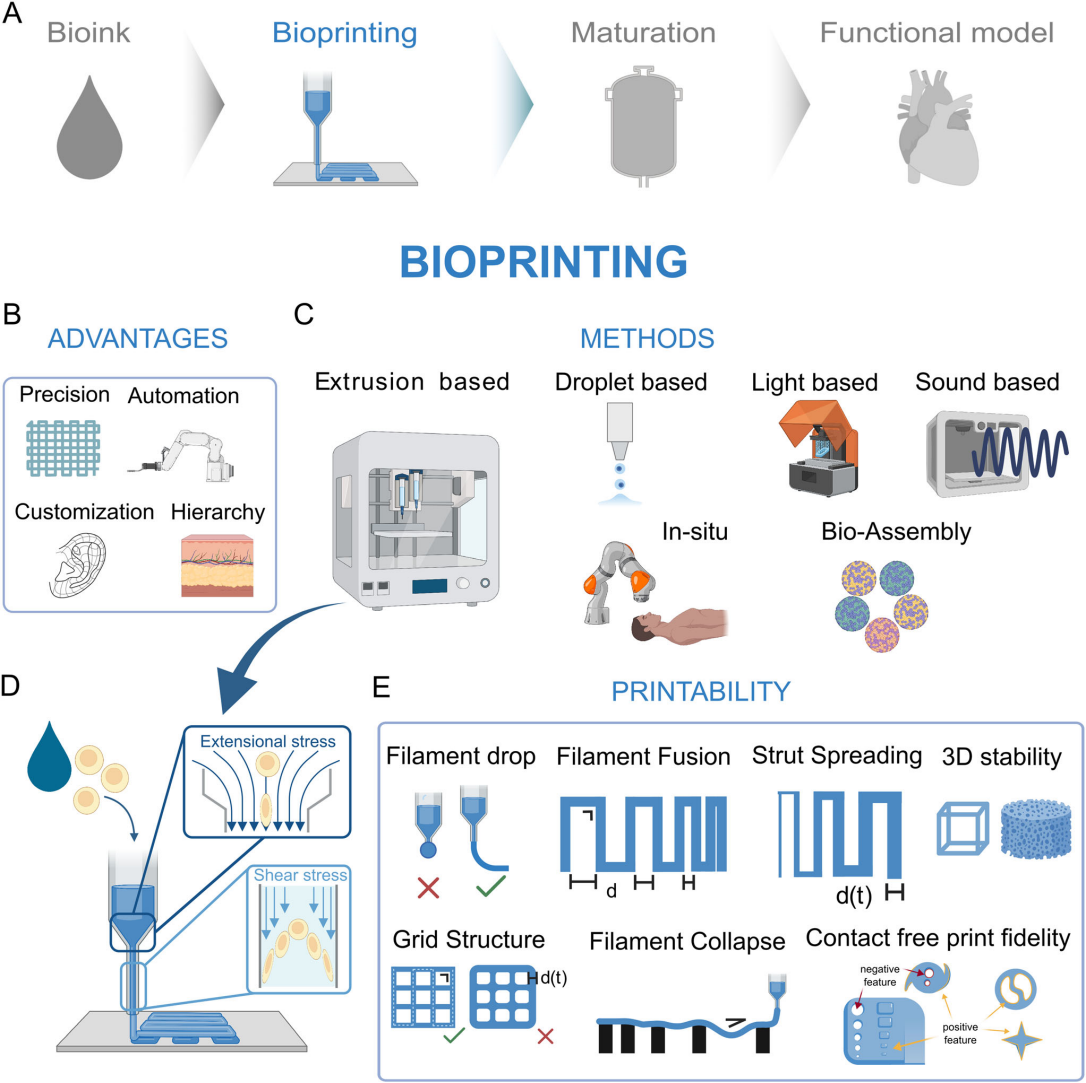
A) Biofabrication workflow: bioprinting. B) The advantages of bioprinting are the resolution of the details and process automation for building customized hierarchical structures. C) Bioprinting involves different methods: extrusion‐based, droplet‐based, light‐based, sound‐based, in situ, and bioassembly. D) In extrusion‐based bioprinting, the inks are pushed out of a nozzle, which induces stress, extension, and shear on the cells. E) To assess the quality of the prints, it is important to keep the geometry and structure intact over time; to measure the printability, some tests can be performed. Created in BioRender. Mussoni, C. (2025) https://BioRender.com/yid0ai3.

After the material is chosen, it must be shaped into the desired tissue geometry by printing (**Figure** **5**A). Currently, machinery has taken over many tasks, and industries are increasingly relying on technology and automation to increase production efficiency. Automated processes offer significant advantages, including higher reproducibility, precision, resolution, versatility, and scalability, which machines can achieve far more effectively than humans can achieve (Figure 5B). Moreover, with the rise of additive manufacturing and 3D printing technologies, the ability to customize and personalize products has accelerated design processes, enabling rapid prototyping and optimization cycles. These benefits, which have transformed various industries, are now being applied to medicine and biotechnology, inspiring the use of automation and technology to produce tissues. This is where bioprinting comes into play; that technology involves the use of automated systems to precisely deposit bioinks layer by layer, following a 3D digital design, as part of an additive manufacturing process. Tissues are highly complex structures that are well organized at different scales, ranging from the submillimeter scale to the centimeter scale, and simple casting often cannot recreate the layering and intricacies of these structures. Therefore, 3D printing technologies are particularly adept at reproducing the hierarchical structures of tissues down to very small resolutions, while the automation of such processes can help with reproducibility and scalability by standardizing production.^[^ ^165^ ^]^ Over 20 years of development in the field have resulted in many different methods for bioprinting, each with its own merits. When deciding what printing technology to use, important aspects to consider are the size of the tissue to be fabricated, the resolution of its parts, the materials to be used, and the geometry to print. The latter two parameters are also related to the mechanical properties that are needed.

From a technological point of view, some improvement are needed for widespread application of bioprinting, such as better commercial printers, smart nozzles, faster processing or higher resolution. Technologies are still in the developmental stage, but their potential is immense, especially when cooperation is strengthened with mechanical and robotic engineering.

#### 2.4.1 Bioprinting Technologies

Recently, many platforms for bioprinting have been developed, broadening the possibilities for the production of tissue‐like structures. An overview of the various bioprinting technologies available is presented (**Table** **2**), with a clear focus on extrusion bioprinting. Other important definitions that are rising in popularity within the field, such as in situ bioprinting and bioassembly, will also be addressed, as they present important strategies in regard to the bioprinting of organs (Figure 5C).

**Table 2:** Overview of the different techniques of bioprinting sorted according to printing technology strategy to form 3D constructs.

Type of Printing	Definition	Resolution	Type of Ink	Ref.
Droplet based				
Inkjet: Drop‐on‐Demand Thermal, Piezoelectric	The bioink solution is forced under pressure through a nozzle, where it breaks up into droplets. Pressure pulses can be generated pneumatically, with a thermal or piezoelectric actuator.	50 ‐ 100 um	Low viscosity ink	[167]
Acoustic droplet ejection	An acoustic radiation force of an ultrasonic field transmits a force to generate droplets of the bioink	10 µm‐1 mm	Low viscosity ink	[221]
Microvalve	Electromechanical valves allow the drop of the bioink when the pneumatic pressure overcomes the fluid viscosity and surface tension at the opening	1‐2 µm	Low viscosity ink	[222]
Laser Induced Transfer	A laser hits the bioink, causing a bubble to form and a droplet is produced. The transfer can be forward, side or backward based on the laser positioning	10‐20 µm	Low viscosity ink	[223]
Droplet Cryoprinting	A stream of bioink droplets generated by microvalve is deposited on a cryoplate, thus freezing.	400 µm	Low viscosity ink	[169]
Microgel Printing	Microgel are generated on a microfluidic chip mounted on moving axes and deposited on a cryoplate, thus freezing	50 µm	Microgels	[170]
Extrusion Based				
Source of pressure: Piston, Screw, Air Pressure	A bioink is extruded through a nozzle and forms a strand that is deposited into a specific design in a layer‐by‐layer fashion. The extruding pressure can be driven by a piston, a screw or pressurized air.	> 100 um	Shear‐Thinning Hydrogel, Multimaterial	[224]
Coaxial	Two or more bioinks are extruded simultaneously through a coaxial nozzle, forming a core strand that is surrounded by one or more shells.	> 870 um	Shear‐Thinning Hydrogel, Multimaterial	[225]
Embedded	A bioink or sacrificial biomaterial is extruded into a viscoelastic matrix that serves as embedding medium. This medium has a solid‐like behavior, gets locally fluidized upon the stress of the printing nozzle and goes back to its solid‐like behavior after extrusion. In this way the extruded ink is trapped into the matrix.	> 10 um	Shear‐Thinning Hydrogel, Multimaterial	[226]
Diffusion Based	The crosslinker is printed in the hydrogel (or vice versa), it diffuses out of the print and the material gets locally crosslinked	100 µm	Shear‐Thinning Hydrogel, Multimaterial	[179]
Cryo printing	The bioink is frozen on the plate upon printing	300 µm	Shear‐Thinning Hydrogel, Multimaterial	[180]
Chaotic Bioprinting	Based on chaotic advection, a laminar flow is created by a static mixer, resulting in multilayered structures.	> 10 um	Shear‐Thinning Hydrogel	[181]
Microfluidic assisted bioprinting	The ink is extruded through a microfluidic chip	10 µm	Hydrogel	[182]
Cell ElectroWriting	An electric field is applied to the print to pull strut and obtain and higher resolution	5‐40 µm	Hydrodynamic hydrogel	[183]
Light Based				
Digital Light Processing (DLP)	A projected light crosslinks a layer of photocrosslinkable resin	10 µm	Photocrosslinkable polymer resin	[227]
Stereolithography (SLA)	Laser cures a layer of photocrosslinkable resin	10 µm	Photocrosslinkable polymer resin	[193]
Volumetric printing	Tomographic projection of light to crosslink a resin	20‐30 µm	Photocrosslinkable polymer resin	[194]
FLIGHT	Focusing the light of volumetric printing, anisotropic structures are formed in the hydrogel	2‐30 µm	Photocrosslinkable polymer resin	[195]
Two Photon Lithography (2PL)	Using InfraRed laser exposure and Ruthenium as a photocatalyst, the bioink is rapidly photocrosslinked layer by layer	128 µm × 128 µm × 10 µm	Photocrosslinkable polymer resin	[228]
Optical Fiber Assisted Printing (OFAP)	An optical fiber locally crosslinks a resin vat	100 µm	Photocrosslinkable polymer resin	[198]
Dynamic interface printing	A hollow print head is open at the bottom and sealed with a transparent glass window at the top. An air–liquid meniscus forms at the print head's end by trapping air when submerged in the bioink. At the meniscus structures are polymerized by visible light transmitted from above.	15 µm	Photocrosslinkable polymer resin	[199]
Xolography	Dual color light‐sheet volumetric printing by moving an ink through a UV light sheet while adapting the focus of orthogonal visible light.	20 µm	Hydrogel‐based photoresin with dual‐color photoinitiator	[196]
Sound Based				
Deep‐penetrating acoustic volumetric printing	A Focused Ultrasound transducer rapidly solidifies the bioink by sonothermal effect	1‐2 mm	Phase‐transition viscoelastic sonicated inks (sono‐ink)	[201]
Acousto‐printing	Ultrasound waves locally induce the crosslinking of a sono‐bioink	1 mm	Ultrasound mediated polymerizing inks	[202]
In Situ				
Bioprinting	All extrusion and inkjet techniques described previously	30‐100 µm	Shear‐Thinning Hydrogel	[204]
Hand‐held	An extrusor moved by hand on the site to cover	1 mm	Shear‐Thinning Hydrogel	[229]
Robotic manipulator	A robotic arm is equipped with a printing technology end effector	200 µm	Shear‐Thinning Hydrogel	[205]
Bioassembly				
Biogripper	The assembloids are trapped and transported to the final spot	size of the assembloids	Assembloids/Microsissues	[230]
Direct printing	Assembloids are printed by extrusion or droplet based printing	size of the assembloids	Assembloids	[213]
Aspiration	Negative pressure is used to fix an assembloid and transport it	size of the assembloids	Assembloids and hydrogels	[231]
Sound	Sound waves organize and pattern the assembloids	size of the assembloids	Assembloids	[218]
Magnetic	Magnetic particles are moved with a magnet and transport the assembloid	size of the assembloids	Assembloids	[232]
Kenzan method	The spheroids are arranged on metal spikes arranged in the desired geometry	size of the assembloids	Assembloids	[220]

##### Droplet‐Based Methods

The first bioprinters established were, in fact, inkjet printers, which are still widely employed. Droplets of a cell‐including low viscosity ink are stream deposited in a controlled geometry.^[^ ^166^ ^]^ This type of printer works by generating droplets via various methods: inkjet, acoustic droplet ejection, microvalve, or laser transfer to be fused together three‐dimensionally layer by layer. The advantages of this technique includes the possibility of bioprinting low‐viscosity bioinks, thus avoiding nozzle clogging, with good accuracy and resolution, up to single cells, and with no contact. The disadvantages are the stability of the constructs and the difficulty associated with noncontinuous printing; this kind of printing is time‐consuming and not suitable for large productions.^[^ ^167^ ^]^

More recent advances in this type of printing include laser‐induced forward transfer,^[^ ^168^ ^]^ where heat generated by a laser projects a bubble in the ink, thus generating a droplet that is transferred to the collector.

One further development of this technique has been to freeze the ink upon deposition on a cryoplate, thus improving the shape retention and vertical resolution caused by the spreading of low viscosity inks.^[^ ^169^ ^]^

A similar technique that could be considered falling under this category is the direct printing of cell‐encapsulating microgels, where a stream of microgels is generated in a microfluidic chip, mounted on moving axes, and deposited according to a specific shape.^[^ ^170^ ^]^

##### Extrusion‐Based

At present, the most commonly used bioprinting technique is extrusion‐based bioprinting.

The most basic technique involves storing a bioink in a cartridge, pushing it out of a nozzle via pneumatic or mechanical forces, and depositing it in a layer‐by‐layer fashion according to the desired geometry.^[^ ^171^ ^]^ The technique has evolved over time to exploit the advantages of alternative designs of basic components, and depending on the type of printer, nozzle, or collector, extrusion‐based bioprinting can be classified into its evolution methods, e.g., coaxial, embedded, and diffusion‐based printing.

Coaxial bioprinting involves a core‒shell concentric and coaxially organized nozzle for the simultaneous extrusion of two or more bioinks, forming a core strand surrounded by one or more shells. The number of concentric layers can vary according to how many concentric nozzles fit the extruder. This technique allows the formation of separated compartments where different cell types or biomaterials with different mechanical orbiological properties can be combined. Coaxial bioprinting is of great interest for producing hierarchically layered or concentric structures.^[^ ^172^ ^]^

In the embedded approach, instead of printing in air, the bioink is extruded in a shear‐thinning support bath to defy gravitational impediments of complex geometries by offering support to the printed filament and allowing the printing of large volumes with high shape fidelity and anatomic accuracy. Notably, the first in‐gel printing method, called freeform reversible embedding of suspended hydrogels (FRESH), involves the use of a thermoreversible hydrogel to support the printing of low‐viscosity biomaterials.^[^ ^173^ ^]^ In alternative methods such as sacrificial writing into functional tissue (SWIFT), the embedding medium is not temporary but consists of cell aggregates, and the printed structures create sacrificial channels resembling vascularized structures with high cellular density.^[^ ^174^ ^]^ Support baths can also be composed of microgels, creating highly porous structures.^[^ ^175^ ^]^

Diffusion‐based approaches have been successfully used for producing multimaterial constructs^[^ ^176^ ^]^ and, most recently, for producing perfusable channels by extruding the crosslinker^[^ ^177^ ^]^ or by interfacial coacervation in aqueous‐two‐phase systems.^[^ ^178^ ^]^ In these approaches, the printed ink diffuses into an embedding medium, and a channel is formed by polymerization at the material interface.^[^ ^179^ ^]^

Owing to the mechanical properties of hydrogels, printing vertically developed structures can be a challenge, which is why cryoprinting was developed. A cryoprotective bioink can be printed vertically on a temperature‐controlled freezing plate. The free‐standing structure presents anisotropic structures typical of ice templating that can help guide cells in tissue formation.^[^ ^180^ ^]^

Another type of extrusion bioprinting leverages the principle of chaotic advection, where a laminar flow is created by a static mixer, resulting in multilayered structures. The final strand composition can be predicted to be formed by multiple compartments and possibly multiple materials.^[^ ^181^ ^]^ This method allows the fabrication of complex microlayered constructs very quickly and can be exploited to generate multiple different interfaces by segregating materials via laminar flow.

When the printhead is equipped with a microfluidic chip as an extruder, it is defined as microfluidic‐assisted bioprinting; in this way, it is possible to achieve high resolution to a scale of tens of micrometres in diameter and to modulate the cell concentration, minimizing cell distress due to the forces in the nozzle.^[^ ^182^ ^]^ Due to the flexibility of the design of chips with multiple microchannels, different combinations and mixes of materials and cells or materials and crosslinkers are also achievable.

Another way to improve resolution and be one step closer to printing the complex intrinsic morphologies of tissues is to apply an electric field while printing a hydrodynamic hydrogel. In electrohydrodynamic bioprinting, the conductive bioink filament is extruded while being pulled by electrostatic forces to achieve strut diameters of 5–40 µm. To date, only a limited number of bioink materials have been successfully bioprinted via this technique, also called “cell electrowriting”; however, other types of biomaterial inks have been printed for tissue engineering purposes in the form of melt electrowriting (MEW).^[^ ^183^ ^]^

##### Melt ElectroWriting

Bioink hydrogels are sometimes not sufficient to recapitulate tissues, especially their mechanical properties, and it is necessary to offer reinforcements with more stable structures or polymers. For this purpose, a nonbioprinting method worth mentioning is MEW.^[^ ^184^, ^185^ ^]^ This fairly stable process is based on the extrusion of a melted thermoplastic polymer, on which a voltage is applied. The electric field pulls the polymer jet to produce very thin (2–20 µm diameter) fibers that can be precisely spatially arranged and even stacked.^[^ ^186^ ^]^ The technique has been used in combination with various forms of bioprinting and has proven to be a valid tool not only for reinforcement but also for enabling cell guidance and obvious tissue layering.^[^ ^187^, ^188^, ^189^, ^190^ ^]^

Following this line of thought, a recent trend of converging multiple bioprinting strategies is to combine the complementary strengths of each method, thus increasing the complexity of the prints and future tissues.

##### Light‐Based

In addition to extrusion‐based bioprinting, other techniques have emerged as alternatives. For example, light‐based bioprinting is taking a lead in the field of biofabrication; light‐based printing foresees the use of light to crosslink a bioresin into the desired shape.^[^ ^191^, ^192^ ^]^ The main advantage of this technique is that it allows for a contactless, fairly rapid print with high resolution (dependent on the pixel dimension). Drawbacks are mostly in the limited processable materials, as the light needs to pass through the resin to polymerize the bioink.

The crosslinking can occur layer by layer with direct light processing, by the digital projection of light and stereolithography,^[^ ^193^ ^]^ or by the projection of a laser, as per standard additive manufacturing techniques. A technique that is on the rise in application is volumetric bioprinting,^[^ ^194^ ^]^ an ultrafast printing method where a tomographic projection of the light crosslinks the resin in a rotating vial in seconds, allowing extremely fast and very detailed printing. In volumetric bioprinting, the light is delivered to the resin by a modulator as a sequence of 2 dimensional (2D) projections of the desired shape, whereas the vial rotates, the sum of the projections activates local polymerization in the irradiated volume where the light dose exceeds the crosslinking threshold.

Its evolution, Filamented Light (FLight), leverages the optical modulation instability of the projected light into beams to print precise anisotropic structures in the shape of filamented channels in the micrometer range.^[^ ^195^ ^]^ Another form of volumetric printing, xolography, which exploits orthogonal UV and visible light to crosslink a photocrosslinkable resin provided with a dual‐color initiator and achieves small resolution, was proven to work for bioprinting cell aggregates^.[^ ^196^, ^197^ ^]^ Additionally, optical fibers have been used in conjunction with a printer to locally and precisely photocrosslink the bio resin and achieve free‐form printed structures.^[^ ^198^ ^]^ A novel and promising publication highlights the combination of light projection and acoustic waves for dynamic interface printing.^[^ ^199^ ^]^ A hollow transparent print head is submerged in a liquid prepolymer solution, trapping air and forming a meniscus. The meniscus serves as an interface where polymerization occurs by patterning light. The meniscus can also be modulated via acoustic waves that modify the pressure at the air‒liquid interface; in this way, the print is not affected by any parameters outside the printhead interface, unlike other kinds or light‐based printers.

##### Sound‐Based

Among contactless technologies, acoustic bioprinting is emerging and in current development for bioprinting applications.^[^ ^200^ ^]^ These methods rely on focused sound waves to locally crosslink bioinks, similar to their light counterparts. Sound waves have deep penetration abilities that are 100 times greater than those of light; thus, they are promising technologies for in situ bioprinting in deep subcutaneous zones.^[^ ^201^, ^202^ ^]^ In one case, volumetric sonic printing is possible by crosslinking a phase transition viscoelastic bioink due to the sonothermal effect of the focused sound waves. The base material used was PEGDA, with the addition of agar microparticles as rheology modifiers, poly(N‐iso‐propylacrylamide) (pNIPAM) as an acoustic absorber and ammonium persulfate as a thermal initiator. This material allows for high‐resolution and fast printing of various geometries of opaque materials and even through centimeter‐thick tissues. Limitations are in the composition of proper bioinks suitable for bioprinting.^[^ ^201^ ^]^ Bioprinting via sonic waves was achieved with PEGDA ink without other additives. This method was used to deliver cells locally within the hydrogel, to deliver drugs and to bioprint, showing polymerization of the bioink without precursors or initiators through tissues.^[^ ^202^ ^]^

##### Other Definitions: In Situ Bioprinting

In most bioprinting approaches, artificial tissues are printed ex vivo, and after maturation is performed, for in situ bioprinting, bioinks or biomaterial inks are directly deposited into or onto the targeted area and undergo maturation within the organism.^[^ ^203^, ^204^ ^]^ This technique has much potential and relevance in both the clinical setting and for emergency treatments. However, it also introduces new complexities that require technical and legal considerations. For example, the printing path needs special examination, as it is most likely a nonplanar surface, requiring specialized planning and integration with imaging or scanning techniques at the printing site. The printheads also need special control feedback loops to avoid damage to the surrounding tissues by accident; in this case, contact‐free techniques may be the most advantageous. Commonly available printers may not be sufficient for this kind of printing, since the level of complexity increases noticeably owing to the anatomical shapes on which to print, instead of a flat collector, or owing to natural body movements such as breathing. For this application, robotic arms or surgical robots equipped with printheads are being developed and could be employed.^[^ ^205^, ^206^ ^]^ Another consideration regarding the materials and shapes is the construct stability and the ability to withstand physiological forces and stresses immediately after printing, before being completely mature. Different techniques, such as inkjet, extrusion‐based, or acoustic bioprinting, have been proven to be promising approaches for in situ bioprinting. Inkjet bioprinting has been used primarily in skin repair, resulting in faster and improved reepithelialization.^[^ ^207^ ^]^ Extrusion‐based printing, whether automated or handheld printers are used and has been used in skin,^[^ ^208^ ^]^ cartilage^[^ ^209^ ^]^ and bone repair.^[^ ^210^ ^]^

##### Bioassembly

To date, top‐down approaches in which bioinks are spatially arranged by the printer have been discussed. However, in the realm of organ bioprinting, another method worth examining is bioassembly: the fusion of semimatured biological building blocks to recreate complex tissues.^[^ ^211^ ^]^ In these building blocks, which are usually spheroids or organoids (aggregates into spheres of one or more cell types), the cells guide histogenesis following the embryonic development of an organ. The 3D environment allows for more complex cell‒cell interactions and more natural cellular behavior, leading to more organized tissue formation. Cellular aggregates can also be engineered to mature in a more controlled manner via biochemical or biophysical cues to guide proliferation, differentiation, and overall maturation. When the aggregates are already partially developed, they can be automatically arranged or printed within a matrix with a specific organized geometry or pattern to recapitulate the tissue hierarchy.^[^ ^212^ ^]^ Over time, the cells within the aggregates start migrating until the blocks fuse together and generate a new, unified, larger tissue. The advantage is the use of high‐density agglomerates of cells, potentially already partially self‐organized, to unite into a larger, high‐cell‐density construct and guide tissue formation by carefully engineering the geometry, organization, and surrounding environment. The basic blocks are assembled and patterned via a variety of techniques, from direct printing of spheroids inside a bioink^[^ ^213^ ^]^ to pick‐and‐place via aspiration^[^ ^214^, ^215^ ^]^ or by guiding the movements and positioning of the agglomerates via magnetic^[^ ^216^, ^217^ ^]^ or acoustic^[^ ^218^ ^]^ forces.^[^ ^219^ ^]^ A very advanced method that has been proven valid is the “Kenzan method”, where spheroids are automatically positioned on an array of nozzles arranged in the construct geometry and stacked to fabricate a scaffold‐free tissue. The spheroids then fused and built a unified tissue.^[^ ^220^ ^]^

#### 2.4.2 Rheology

In extrusion bioprinting, a bioink must flow efficiently through the nozzle while maintaining its intended shape after deposition. This requires careful optimization of the rheological properties of the bioink to ensure both printability and cell viability within the printed construct.^[^ ^233^ ^]^ Both printing resolution and cell survival significantly depend on flow behavior. Good printing resolution is achieved when the bioink can be extruded through a thin nozzle and solidified immediately afterward. On the other hand, cell survival is heavily dependent on the shear forces experienced during printing. In both cases, for example, shear‐thinning behavior is advantageous. To systematically investigate these rheological material properties, rheology—the study of how materials deform and flow—is essential for understanding the behavior of bioinks under extrusion through a nozzle, which typically ranges from 0.1–1 mm in diameter.

For a hydrogel to be successfully printed into a stable structure, it must undergo at least one phase transition. The initial state of a bioink in the cartridge can vary: it may be formulated as a low‐viscosity precursor solution that exhibits shear‐thinning behavior during extrusion, or it can be a preformed physical hydrogel that liquefies only when subjected to the shear stress of the printing nozzle. Crucially, regardless of their initial rheological properties, the printed filaments from both types of bioinks must then undergo rapid postextrusion gelation or resolidification to establish structural integrity instantly and maintain their deposited shape. The viscosity of the bioink in its flow state plays a crucial role in this process. Viscosity, defined as the resistance of a fluid to flow under applied force, directly affects print fidelity, where higher viscosities improve structural integrity but also increase shear stress, which can be detrimental to embedded cells.^[^ ^234^ ^]^ The molecular weight and concentration of polymers in a solution are key factors influencing viscosity.^[^ ^235^ ^]^ Bioinks for extrusion bioprinting typically exhibit viscosities ranging from 30 to 6 × 10⁷ mPas.^[^ ^236^ ^]^ Most bioinks are shear‐thinning and thus non‐Newtonian, allowing them to flow more easily under stress while regaining structure after deposition. Newtonian fluids maintain a linear relationship between shear stress and shear rate, whereas non‐Newtonian fluids exhibit shear‐dependent viscosity, which can be time independent (e.g., shear‐thinning or shear‐thickening) or time dependent (e.g., thixotropic or rheopectic).^[^ ^235^ ^]^

To assess printability, rheological testing is primarily conducted via rotational rheometers equipped with parallel‐plate or cone‐plate geometries.^[^ ^237^ ^]^ The cone‒plate system provides precise measurements of shear stress and shear rate, making it ideal for yield stress, creep, recovery, oscillation, and ramp tests. However, when fillers are incorporated, they are limited to small particle sizes (≤10 µm). In contrast, the parallel‐plate system accommodates larger particles (≥10 µm) but introduces shear rate variations across the sample, requiring careful interpretation when comparing results.^[^ ^238^ ^]^ Key rheological parameters measured in bioinks include viscosity (Pa·s), shear stress (Pa), which represents the force applied per unit area, and shear rate (s^−1^), which describes how velocity changes with distance. The primary method for hydrogel characterization is small‐amplitude oscillatory shear (SAOS), which is used to evaluate viscoelastic properties. In SAOS, a small torsional oscillation generates shear flow, and in a stress‐controlled rheometer, the input follows τ(t) = τ_o_*sin(ωt), whereas the output is γ(t) = γ_o_sin(ωt+δ). The phase shift δ indicates the material's behavior, where δ = 90° corresponds to a purely viscous material and δ = 0° corresponds to a purely elastic material. The complex shear modulus (G*) is expressed as G* = G′+G′′, where G′ (storage modulus) represents the elastic behavior and stored energy, whereas G″ (loss modulus) describes the viscous behavior and energy dissipation. The loss tangent (tan δ = G″/G′) serves as an indicator of material behavior, with tan δ>1 indicating a more viscous material and tan δ < 1 indicating a more elastic material.^[^ ^239^ ^]^ A crucial aspect of bioink testing is amplitude sweeps, where the strain amplitude increases at a constant frequency to determine the linear viscoelastic (LVE) region. Within this region, the material exhibits reversible deformation, meaning that G′ and G″ remain independent of the strain amplitude. As the strain increases beyond the yield point, the material enters a nonlinear regime, where structural changes, such as polymer disentanglement or bond rupture, cause G′ to decrease, indicating a transition to a more fluid‐like state. Amplitude sweeps are essential for identifying material stability under deformation and the onset of shear‐induced breakdown.

Similarly, frequency sweeps play a key role in analyzing the time‐dependent behavior of hydrogels. In this test, the oscillation frequency increases at a constant strain amplitude, allowing the storage modulus (G′) and loss modulus (G″) to be plotted as functions of frequency. Frequency sweeps provide insight into the molecular architecture, network crosslinking density, and relaxation dynamics of the hydrogel. As the strain increases beyond the yield point, the material enters a nonlinear regime, where structural changes, such as polymer disentanglement or bond rupture, cause G′ to decrease, indicating a transition to a more fluid‐like state. On the basis of these measurements, the yield stress can also be determined, which needs to be overcome to induce material flow. By monitoring G′ and G″ as a function of temperature, gelation and melting points can be determined, ensuring that bioinks maintain structural stability within physiological conditions. To quantify the crosslinking kinetics of precursor solutions during gelation, time sweeps can also be performed. In these measurements, both the strain amplitude, which remains at very low levels within the LVE  region, and the frequency are held constant to minimize mechanical disturbances during the monitoring process. Additionally, time sweeps can be conducted immediately following an amplitude sweep that temporarily liquefies the material to assess the shear recovery—the time the material requires at rest to re‐establish its gel network. This recovery behavior is a critical parameter for print resolution, as it reflects the postextrusion structural recovery of the bioink. At low frequencies, viscous behavior dominates, whereas at high frequencies, elasticity increases because of limited molecular mobility. The crossover point (ω_c_), where G′ and G″ intersect (tan δ = 1), is a valuable indicator of the hydrogel relaxation time (τ) and provides a measure of dynamicity and network stability.^[^ ^240^ ^]^ In addition to SAOS, temperature sweeps assess the thermal responsiveness of bioinks, particularly in thermosensitive hydrogels such as GelMA or agarose. By monitoring G′ and G″ as a function of temperature, gelation and melting points can be determined, ensuring that bioinks maintain structural stability within physiological conditions.^[^ ^241^ ^]^

In addition to their base material properties, fillers, crosslinkers, and cells significantly influence bioink rheology.^[^ ^242^ ^]^ Additives can modify shear‐thinning properties, alter postextrusion recovery, and impact network integrity.^[^ ^243^ ^]^ The presence of biological cells introduces additional complexity, as cell‐laden bioinks may behave differently from their acellular counterparts due to cellular interactions, matrix remodeling, or mechanical feedback. Consequently, rheological assessments must be conducted on both the base bioink and the final formulation, ensuring that the material maintains optimal flow properties, viscoelastic behavior, and printed structure stability under bioprinting conditions.

#### 2.4.3 Hydrodynamic Forces in Bioprinting Processes

A comprehensive examination of the role of mechanical stress in extrusion‐based bioprinting should consider the kinds of stresses occurring within the hydrogel and how they affect cells (Figure 5D). During the entire bioprinting process, hydrodynamic shear stress and extensional stress are the two relevant forces affecting cells.^[^ ^244^ ^]^ Small shear stresses already occur during bioink mixing and transfer into the nozzle, but most relevant is the shear stress applied to cells while they pass through the narrow nozzle tip. Extensional stress emerges in the area of abrupt velocity changes at the transition to the nozzle tip, where the cross‐sectional area of the nozzle is suddenly contracted.^[^ ^245^ ^]^ Both types of stresses affect the biochemistry of cells. The cellular mechanoresponse can include cytoskeletal rearrangement, changes in protein expression and cellular motility, as well as cell growth, proliferation, differentiation or death.^[^ ^246^ ^]^ In 2009, one of the first methods to model cell damage during that time, the “biodispensing” process, was presented. The authors reported that the shear stress and exposure time in the nozzle played a major role, whereas the hydrostatic pressure had only a small effect on the cells.^[^ ^247^ ^]^ The effects of the nozzle geometry on the flow rate and degree of cell damage have also been studied extensively.^[^ ^248^, ^249^, ^250^ ^]^ Cell viability increases with decreasing nozzle length, increasing nozzle diameter or the use of conical instead of tubular nozzles.^[^ ^251^ ^]^ In conical nozzles, both shear and extensional stresses are lower than those in cylindrical nozzles. Conical nozzles require a lower printing pressure than cylindrical nozzles do to reach the same flow rate, resulting in better cell viability.^[^ ^252^ ^]^ Regarding bioink properties, it was found that for a Newtonian ink, the shear force on cells increases with ink viscosity.^[^ ^253^ ^]^ In contrast, shear‐thinning bioinks have reduced viscosity under shear strain, leading to less shear stress exposure for the cells. Stress‐induced damage to the membrane due to extrusion bioprinting can be reduced with the supplementation of calcium in bioinks to facilitate the resealing of damaged sites.^[^ ^254^ ^]^ Another way to increase cell viability is to encapsulate different cell types within alginate hydrogels to protect them from damaging forces, which significantly improves viability.^[^ ^255^ ^]^ Recently, cell‐encapsulating microgels have attracted considerable interest because they not only protect cells during the printing process but also control the cellular microenvironment. With increasing knowledge about the mechanical forces that impact cells during the bioprinting process, they can also be effectively considered. For example, the shear force in the nozzle can be used for the controlled alignment of cells or fibers.^[^ ^256^ ^]^

#### 2.4.4 Printability Assessments

As extrusion‐based bioprinting is the main process currently used to fabricate artificial tissues, it is important to establish common evaluation parameters to ensure the appropriate printing quality of the construct. Each bioprinting technique illustrated in combination with different materials has its own merits and issues in regard to bioprinting constructs. After printing, it is always necessary to evaluate the state of a construct in relation to the initial design and desired properties to establish if the processing of the material has been successful for the application. For each printing method, different parameters can be evaluated to establish whether a print is good; however, it is important to establish common evaluation parameters across the field to ensure the appropriate and consistent printing quality of the construct.

Printability is a property defined as the ability of a bioink to be flowable, allowing it to be precisely deposited with good spatial, temporal and volumetric control.^[^ ^257^ ^]^ To successfully print the desired geometries and structures that closely mimic native tissue, the fundamental properties to consider are the extrudability, resolution, shape fidelity and stability of the prints (Figure 5E).  Extrudability refers to the investigation of a set of parameters that solely allow extrusion of an ink from the nozzle, ^[^ ^235^ ^]^ which is also related to the rheological properties. Resolution is a technology‐dependent property, and it is the smallest unit that can be printed. Shape fidelity is analyzed by comparing the printed geometry with its digital counterpart (e.g., CAD). In addition, the printed constructs aim to withstand the time and effects of maturation, which corresponds to stability.

In efforts to guarantee standardization and comparability, several tests have been developed to assess the printability of bioinks and the structural integrity of the prints. The filament drop, filament fusion, strut spreading, grid structure and filament collapse tests were performed.^[^ ^235^, ^258^, ^259^, ^260^ ^]^ Filament drop tests the formation of a filament upon extrusion, as opposed to droplets. Filament fusion analyses the print of parallel lines gradually moving closer to each other until they merge. Strut spreading investigates the printing of parallel lines with gradually increasing diameters due to pressure, whereas the grid structure tests the printing of perpendicular lines to form a multilayered grid. The filament collapse test observes the behavior of one strut printed over columns with increasing spacing. Recent efforts in improving print quality have utilized live imaging during the printing process to correct the extrusion of the bioink online. Algorithms or artificial intelligence analyze the images of the deposited strut and guide the printing parameter correction in real time.^[^ ^261^, ^262^ ^]^ Interestingly, the results from printability tests can be simultaneously correlated with the rheological properties of the bioink.^[^ ^105^ ^]^ Recent improvements in the imaging, optical assessment, and standardization of such assays have also improved the rate, reliability and comparability.^[^ ^258^ ^]^ Furthermore, machine learning has already been used to better understand the printability of an ink on the basis of rheological measurements.^[^ ^263^ ^]^ Other techniques, such as light‐based methods, which have the advantage of very high resolution, need different evaluation criteria than extrusion‐based methods do, as they work on different principles. The quality of a print is often verified by printing specifically designed constructs presenting positive features, e.g., spikes, pillars or negative features, e.g., holes, as well as convex or concave shapes.^[^ ^196^ ^]^ Beyond custom shapes with triangles and circles, gyroids are often chosen as complex geometries that can highlight the printer's abilities.^[^ ^264^, ^265^, ^266^ ^]^

In addition to defining the resolution that can be achieved by the printer, the 3D stability of the printed material also needs testing; therefore, hollow cubes or pyramidal structures are tested to verify how the printed resin can withstand gravity.

Notably, the technical and environmental conditions during printing also affect the print quality. The technical limits include the printer hardware, cartridge or nozzle size, room temperature, nozzle temperature and sometimes humidity, especially when dealing with thermally responsive inks such as gelatin‐based inks. Another factor to consider is the surface onto which the printing is conducted, which may influence the ink performance. For example, glass and plastic surfaces do not yield the same results in some cases because of their different surface hydrophobicities.

### 2.5 Maturation of Bioprinted Fabricates

**Figure 6:**
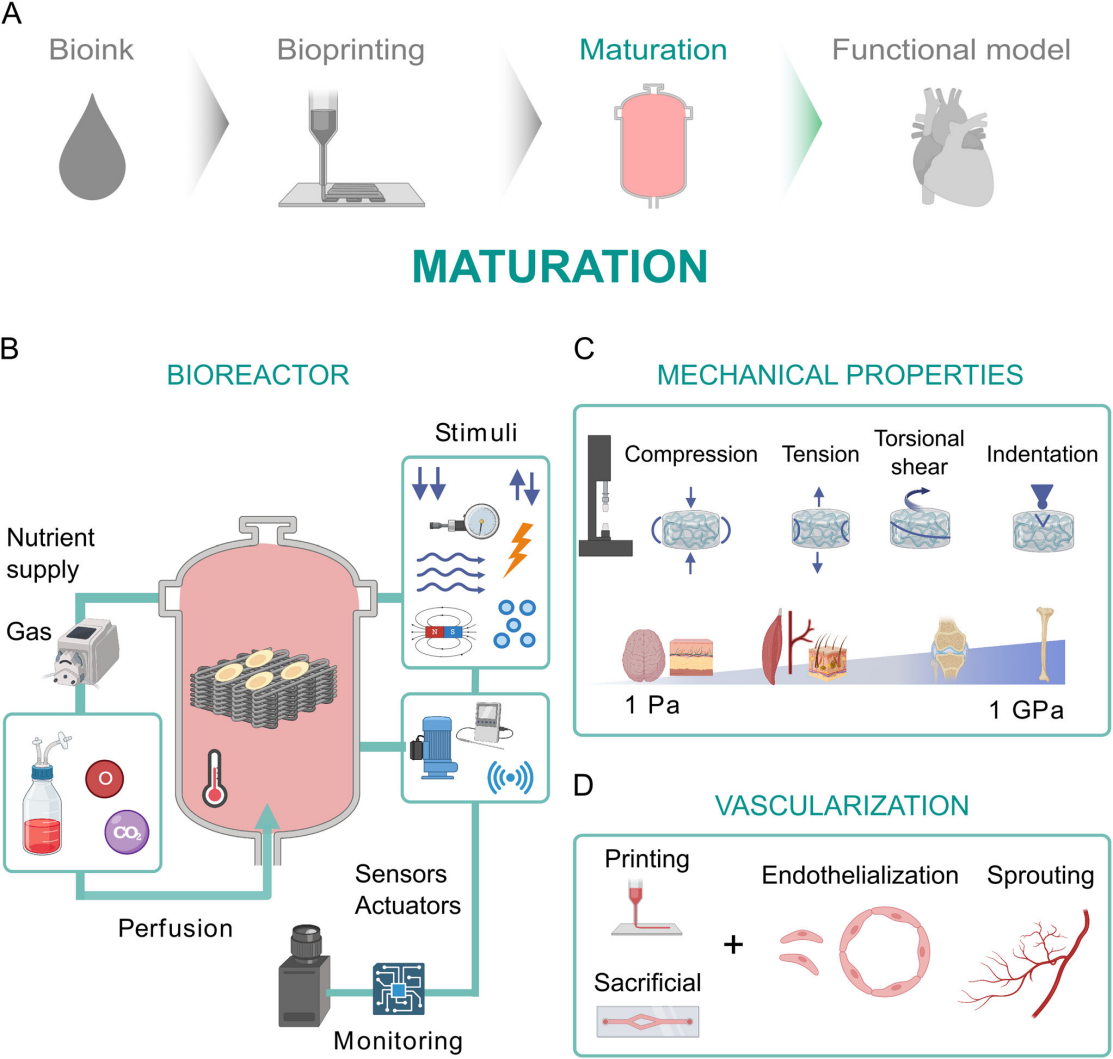
A) Biofabrication workflow: maturation. B) The construct matures in a bioreactor where it is subjected to stimuli of different natures and monitored in a controlled environment. C) A very important aspect of artificial tissue is its mechanical properties, which should match those of native tissue. D) The key for tissue survival is nutrient transportation via diffusion or by incorporating the vasculature into the tissue. Created in BioRender. Mussoni, C. (2025) https://BioRender.com/yid0ai3.

After a construct is bioprinted, it is not yet ready and functional; it needs time to mature into the desired tissue (**Figure** **6**A). The first challenge is the survival of larger tissues with the transportation of nutrients to the core of prints, especially if bulky. Second, the functionality of the tissue needs to be developed, which means that cells are actively fulfilling their duties and competences, remodeling the bioink and producing the correct signals and molecules; to do so, they need to be exposed to appropriate environmental signaling. It is important to mimic physiological conditions during culture to favor cell growth, differentiation, and organization, which is possible, for example, in bioreactors. While the human body is the best bioreactor, technology can help to artificially recapitulate conditions comparable to those in native tissues, and this is the first important challenge regarding the postprocessing of bioprinting.^[^ ^267^ ^]^

#### 2.5.1 Bioreactors and Maturation

The fundamental task of a bioreactor is the delivery of nutrients and oxygen to keep the tissue alive (Figure 6B). Media circulate by rocking or mixing the liquid or actively running pumps (peristaltic or linear); oxygen can be generated in a sterile manner by an oxygenator or by exposing media to air. In recent years, efforts have been made to include other strategies for oxygen production close to the scaffolds, including the use of oxygen‐generating particles.^[^ ^268^ ^]^ All of these efforts ensure the longevity of the print, but unfortunately, they are often not enough for the development of fully functional tissue. Hydrogels provide a great temporary matrix for cells. However, it is necessary to provide specific cues to direct cell maturation. Examples of such cues are shear stresses, pressure, forces, mechanical or chemical gradients, growth factors, electrical or magnetic stimuli, and air‒liquid interfaces. To expose the tissue to stimuli, these systems include actuators, pistons, and electrodes for probing, moving, and stimulating the construct.^[^ ^269^, ^270^, ^271^ ^]^ Optical or biochemical sensors can be integrated to online monitor key parameters, such as oxygen, pH, glucose, flow, and pressure, but other parameters, such as amino acids and growth factors, should also be considered for monitoring.^[^ ^272^, ^273^ ^]^ These sensors can also be used to regulate the inputs to keep cultures in optimal conditions.^[^ ^274^ ^]^ In addition, a bioreactor should simultaneously image the maturing biofabricates to ensure proper growth and development. Therefore, the current challenge is parallel live imaging for in situ assessments.^[^ ^275^ ^]^

For example, muscle tissue needs electrical stimuli to stimulate contraction, which is very important for its physiology, while measuring the forces from stretching can provide valuable feedback for it status;^[^ ^276^ ^]^ for bone, mechanical compressions emulating the loads from movements on the tissue will improve maturation.^[^ ^277^ ^]^ Blood vessel flow and pressure are fundamental to emulating in vivo conditions on the cells, and they can be modulated to be constant or pulsatile depending on the desired level of complexity.^[^ ^278^ ^]^ In lungs cells are continuously exposed to the air‒liquid interface, and the tissue stretches in cycles with breathing; therefore, a bioreactor should exert air pressure on the tissue and circulate the media.^[^ ^279^ ^]^

The maturation of constructs can take up to months, therefore the financial aspects of such cultures should not be underestimated. Therefore, one complementary approach to reduce time and cost is the optimization of culture conditions by using computational models.^[^ ^280^ ^]^ Such models allow us to evaluate the effects of the bioreactor on the structure in silico to determine that all forces, flows and pressures are optimal.^[^ ^281^, ^282^ ^]^ Beyond microenvironment systems for larger constructs, such as organs‐on‐a‐chip, the current limitation of bioreactors is the need for complex and intricate machinery that should also preserve the sterility of the environment and maintain functionality. Issues such as contamination, clogging or lack of gas (particularly oxygen) distribution are very common and lead to complete failure of maturation. These systems often take up much space, require dedicated incubators, and require significant time and effort to maintain. Many groups and companies are thus working on automated systems, concurring with the rapid development of cultured 3D tissue.^[^ ^283^ ^]^

#### 2.5.2 Mechanical Properties of the Tissues

The mechanical properties of the printed tissues play a vital role in material design and application in tissue engineering. Depending on the application, tissue surrogates must meet certain mechanical requirements under various loading modes (e.g., compression, tension, and shear) to ensure the same functionality of the native tissue (Figure 6C). This applies not only to tissues with clear mechanical functionality, such as articular cartilage but also to any tissue in the human body because cells sense and respond to their mechanical environment and develop their proper functionality only when the microenvironment resembles the native tissue properties.^[^ ^284^, ^285^ ^]^ Human tissues exhibit stiffnesses that vary from 1 kPa in fat tissue and brain tissue to tens of GPa in bones, with all values in between; for example, for the kidney, the stiffness is 5–10 kPa, whereas that of cartilage is ≈1 GPa.^[^ ^286^ ^]^ Furthermore, studies on human tissues such as the brain^[^ ^287^, ^288^ ^]^ and articular cartilage tissue^[^ ^289^ ^]^ have shown that native tissue properties are characterized by nonlinear, time‐dependent (hysteretic) behavior under large deformations, highlighting the complexity of tissue mechanics. This underlines the necessity of multimodal large‐strain mechanical assessments to characterize the highly complex mechanical response of native tissue fully and to design tissue replacements, which reconstitute the mechanical behavior more closely.

Bioprinting is an advantageous technique for recreating tissue organization and hierarchy. This technique allows the reproduction of mechanical gradients, anisotropy, or areas with different properties, owing to the freedom of geometry design and versatility in the material´s spatial arrangement. With proper material selection and scaffold design, we can ensure an appropriate environment to stimulate the printed tissue models to mimic the mechanical behavior of native tissue throughout the formation of a functional ECM. In fact, maturation of the printed organs foresees that cells constantly remodel the surrounding tissue and substitute a biodegradable artificial matrix with a naturally self‐produced matrix, thus changing the mechanical properties of the tissues over time.^[^ ^290^, ^291^ ^]^ The investigation of postprocessing properties is not trivial but important, as in cases of implantation, it could prove to be the difference between a successful result or failure.^[^ ^292^ ^]^

#### 2.5.3 Vascularization

The production of artificial vasculature remains one of the greatest challenges in the field of biofabrication. It is of vital importance to ensure the transport of nutrients, gas exchange into the surrounding tissue and maturation of the prints and thus limits the upscaling possibilities of viable tissues.^[^ ^293^ ^]^ To achieve an optimal supply to larger tissues, vascularization can be mimicked via different strategies. On the one hand, it is possible to print larger cellularized main vessels (mm in size) and induce the sprouting of smaller vessels into the surrounding tissue; on the other hand, microsized vessels can be directly printed into the tissue model^[^ ^294^ ^]^ (Figure 6D).

From a processing perspective, the channel structure of vessels can be formed via direct printing of the material in a tubular shape or created via evacuation of sacrificial materials. Finally, endothelial cells can also come into play at various time points during the fabrication process, either directly during printing or in the form of postseeding channels. The additional presence of supportive stromal cells such as fibroblasts or pericytes is known to stabilize the formed capillaries and contribute to vessel development and maturation.^[^ ^172^, ^295^ ^]^ It is therefore a common strategy to either combine these cell types or include supportive cells in the surrounding matrix. After fully covering the main channel in a tight monolayer, endothelial cells are incentivized by nature to sprout outside the main capillary and spread into the surrounding tissue in a process known as angiogenesis. Thus, the chosen material needs to favor cell migration.

The perfusability of channels is fundamental for keeping the artificial lumen open, and the presence of flow during maturation is one of the key environmental cues that direct endothelial cells towards a functional phenotype, support their proliferation and stimulate angiogenesis. Additionally, ensuring laminar flow is essential for cell alignment. Perfusion during culture can be achieved by using microfluidic peristaltic pumps.^[^ ^296^, ^297^ ^]^

The bioprinting of larger vessels will be discussed later, while regarding the production of artificial blood vessels in the micrometer range, such as small arteries and veins, venules, arterioles and capillaries, different approaches have the potential to fabricate vascular channels. Coaxial printing is often used with sacrificial materials, such as gelatin, Pluronic F127, or Polyvinyl alcohol, as the core of the structure for channel generation and bioink hydrogel as a shell layer. This allows the deposition of endothelial cell‐loaded gels around the sacrificial structure to create the inner endothelial monolayer typical for blood vessels, resulting in good overall cell viability and elongation.^[^ ^298^, ^299^, ^300^ ^]^ Nevertheless, this approach is limited to single strands. An alternative is to fabricate sacrificial interconnected structures that can be embedded in hydrogels and subsequently seeded with vascular cells to produce vascular networks.^[^ ^174^, ^177^, ^301^ ^]^ The materials used for channel generation are PCL, pNIPAM, Pluronic, polyvinylacetate, gallium^[^ ^302^, ^303^ ^]^ or the thermoresponsive polymer PcycloPrOx,^[^ ^304^ ^]^ which shows great potential as a sacrificial material because of its great processability with MEW, allowing the production of thin fibers (16 µm in diameter). By embedding these materials into a hydrogel and subsequently removing the polymer, perfusable complex channel architectures in 2D and 3D are created. The channels are then seeded with endothelial cells, which form a confluent monolayer.

Most recently, the field of vascular biofabrication has tended toward direct printing of endothelial cells in the lumen, also known as in situ endothelialization. Some examples include the use of interfacial coacervation,^[^ ^178^ ^]^ coaxial bioprinting into functional tissue^[^ ^305^ ^]^ or the use of smart materials that dissolve after printing and can be used as sacrificial cell‐laden bioinks.^[^ ^306^, ^307^ ^]^

Challenges in the vascularization of tissues lie in the scalability of the process, the perfusion and connection of networks of different sizes to the bioreactors and intentionally guiding the process of angiogenesis.^[^ ^308^ ^]^

#### 2.5.4 Scaffold Degradation and ECM Remodeling During In Vitro Maturation

A critical yet often underappreciated factor in the in vitro maturation of bioprinted constructs is the degradability of the employed bioinks. Many hydrogel‐based bioinks, particularly those derived from natural polymers such as collagen, fibrin, and hyaluronic acid, are susceptible to enzymatic degradation through cell‐secreted proteases such as matrix metalloproteinases (MMPs) or fibrinolytic enzymes.^[^ ^309^, ^310^, ^311^, ^312^ ^]^ In addition, hydrolytic degradation processes affect chemically modified polysaccharides such as oxidized alginate.^[^ ^313^, ^314^ ^]^ These degradation mechanisms are essential for allowing cells to actively remodel their microenvironment, deposit tissue‐specific ECM, and progress toward a functional phenotype.^[^ ^315^, ^316^ ^]^ Importantly, the rate and mode of degradation must be carefully tuned to match ECM production and mechanical demands. Too rapid degradation can lead to structural collapse before sufficient tissue has formed, whereas too persistent scaffolds may hinder cellular activity and prevent remodeling.^[^ ^317^ ^]^ Several strategies have been proposed to balance these dynamics, such as the use of hybrid scaffolds with dual‐phase degradation kinetics^[^ ^318^ ^]^ or the incorporation of stabilizing fillers such as silk nanofibers or calcium silicates, which support load‐bearing functionality during the early stages of maturation.^[^ ^319^, ^320^ ^]^ Crosslinking chemistry plays a key role here, with enzymatic systems offering bioresponsive remodeling potential, whereas orthogonal or multimodal chemistries enable tighter control over degradation and mechanical properties.^[^ ^321^, ^322^, ^323^ ^]^ The degradability of bioinks is not only relevant for the biochemical and structural adaptation of the forming tissue but also closely linked to the physical microenvironment. In bioreactors, where cells are exposed to mechanical loading and flow conditions, scaffold degradation can be modulated by enzymatic activity and mechanical stress. Delayed degradation of sacrificial phases, for example, has been exploited to generate temporary support structures that dissolve as tissue forms,^[^ ^324^ ^]^ whereas magnetically or mechanically stimulated bioinks promote faster matrix remodeling and tissue‐specific differentiation under dynamic culture conditions.^[^ ^325^ ^]^ Ultimately, the design of degradable bioinks must aim to synchronize scaffold resorption with the formation of a functional ECM. This requires the integration of degradation profiles, crosslinking strategies, cell types, and culture environments into a unified system. Computational models and in situ imaging tools are increasingly employed to predict and monitor these interactions, offering promising approaches to rationally guide tissue maturation in vitro.^[^ ^326^, ^327^, ^328^, ^329^ ^]^ Addressing the complex interplay between scaffold degradation and ECM deposition is thus essential for advancing next‐generation biofabrication strategies.

## 3 Advances in the Biofabrication of Functional Tissues

**Figure 7:**
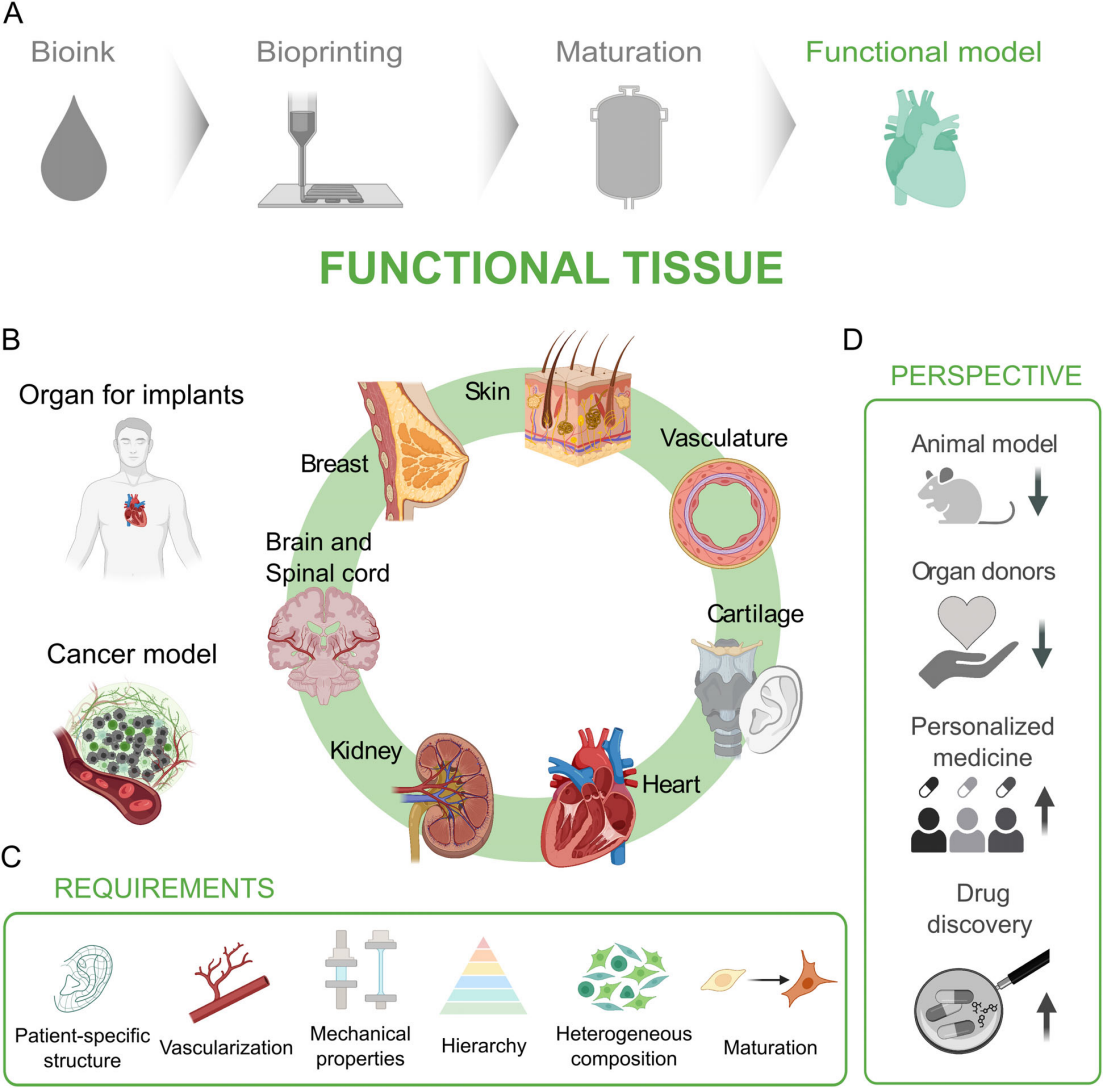
A) Biofabrication workflow: functional tissue. B) Representative functional models for various organs—including the skin, vasculature, cartilage, heart, kidney, brain and spinal cord, and breast—illustrating applications as organ implants and cancer models. C) Requirements for those models are depicted in the lower left lane (patient‐specific structures, vascularization, mechanical properties, hierarchy, heterogeneous composition, and maturation). D) Perspective: decrease in animal models and organ donors while driving advancements in drug discovery and personalized medicine. Created in BioRender. Mussoni, C. (2025) https://BioRender.com/yid0ai3.

Once the bioprinted constructs mature and cells start expressing their function, the tissue is ready for its final purpose. (**Figure** **7**A) They can serve diverse applications, functioning either as models for research and drug testing or as tissues and organs intended for transplantation. (Figure 7B). In general, those constructs must meet key requirements: patient‐specific anatomy, vascularization, mechanical integrity, hierarchical and heterogeneous organization, and proper maturation (Figure 7C). Fully functional tissue fulfills all of these requirements, closely replicating the native counterpart to restore complete physiological function. However, functional tissue meets these criteria only partially, providing enough structure and biological activity to support some intended functions but not yet matching the full complexity or performance of native tissue.

The bioprinting of functional tissues is still in its early stages. However, great strides in technological development have enabled the creation of complex tissue and organ models.^[^ ^330^ ^]^ Nevertheless, issues such as vascularization, scalability and cellular maturation remain prevalent throughout the field of biofabrication.

Advancing organ biofabrication could ultimately reduce reliance on organ donors in the future. In this respect, major challenges occur, starting from the total size of the organ and the complexity of the multiple structures that are aimed to be developed. In addition to whole organ models, there are strategies for the creation of disease models designed to mimic pathological conditions, such as cancer, fibrosis, neurodegenerative disorders, and cardiovascular diseases. Tissue models are currently the primary application of bioprinted constructs, resulting in powerful tools to potentially reduce reliance on animal testing, enhancing the efficiency of drug discovery and incorporating patient‐derived cells, paving the way for affordable personalized medicine (Figure 7D). Biofabricated models of diseased functional tissue have reached a level of contention with organoid and organ‐on‐chip models, which are currently the gold standard.^[^ ^331^, ^332^, ^333^ ^]^ For this reason, bioprinted constructs are ready to reach the stage of clinical relevance as technology continues to advance. They can be used for drug testing or the enhancement of mechanistic disease understanding and are thus poised to play a pivotal role. Bioprinted models offer a unique in vitro platform that closely mimics the cellular, mechanical, and chemical aspects of tissues in a physiological environment. Standardization, reproducibility and high throughput are fundamental next steps for bioprinting as a whole. In addition, earning the trust of the medical community is a short‐term goal that needs to be achieved for bioprinted constructs.^[^ ^334^ ^]^

In the following, we summarize the state of the art of bioprinting for exemplary types of tissues, i.e., skin, cartilage, kidney, heart, brain, spinal cord, and breast, as well as some disease models. In this context, we also highlight the structural and functional requirements of these tissues and models as well as the typically used materials, cells, and promising applications of biofabrication (**Table** **3**).

**Table 3:** Overview of the state of the art in bioprinting for various organs, highlighting the structural and functional requirements of these tissues, commonly used materials, and cell types, as well as their applications.

Organ	Printing Technology	Biomaterials	Cells	Maturation	Functionality	Application	Ref
Vasculature							
Channel diameter 90–300 µm	MEW of Sacrificial material	POx ‐ GelMA	HUVECs	Perfusion	Markers (ICC:CD31), Barrier function, endothelialization, response to inflammation	Vascularization	[304]
1 mm	InGel diffusion printing	GelMA	HUVECs, RFP‐HUVECs, GFP‐HUAEC	Static Media	Endothelialization, barrier, perfusion	Vascularization	[307]
4 mm	Extrusion based printing	GelMA‐PEGDA and nanosilicates	HUVECs, VSMCs	Static Media	Endothelialization	Tissue Model	[447]
4 mm	Extrusion based printing	GelMA, gelatin, HA, Glycerol	HUVECs, HASMCs	Static Media	Endothelialization, Mechanical Properties, cell distribution	Implant	[448]
0.9–2 mm	Triple coaxial printing	dECM (vascular), Alginate	HUVECs, HASMCs	Static Media, pulsatile flow, in vivo (rat)	Endothelialization, mechanical properties, flow, systolic velocity	Implant	[345]
0.3–1.5 mm	Embedded‐Coaxial Printing	Alginate, Gelatin, Collagen	HUVECs, HASMC, BJFF iPSCs	Perfusion	Barrier function, drug response	Vascularization	[305]
3 mm	Droplet Based	Agarose, Alginate	L929	Static Media	Viability	Tissue model	[346]
3 mm	MEW‐electrospinning	PCL, PEU	ECFCs, MSCs	Static Media	Endothelialization	Implant	[340]
1–3 mm	MEW‐Volumertric Printing	PCL, GelMA	GFP‐HUVECs, hbMSCs	Static Media	Mechanical Properties	Tissue model	[188]
1.5 mm	Bioassembly (Kenzan)	Scaffold free	HUVECs, HASMCs, HNDFBs spheroids	Perfusion, In vivo	Anatomy, perfusability	Implant	[347]
ECs: endothelial cells; HUVECs: human umbilical vein endothelial cells; HUAVECs: human umbilical artery endothelial cells; RFP_HUVECs: Red Fluorescent protein‐expressing HUVECs; GFP‐HUAECs: greeen fluorescent protein‐expressing HUAECS; VSMCs human umbilical artery smooth muscle cells; HASMC: human aortic smooth muscle cells; BJFF iPSCs: human fibroblasts induced pluripotent stem cells; L929: mouse fibroblasts; ECECs: endothelial colony forming cells; MSCs: multipotent mesenchymal stromal cells; hbMSCs: human bone marrow derived mesenchymal stromal cells; HNDFBs: human normal dermal fibroblasts.							
Skin							
Epidermis‐Dermis‐Hypodermis	Extrusion based bioprinting	Fibrinogen	hKTCs, human dark melanocytes, dermal hFBs, FDPCs, DMECs, human preadipocytes	Static media, in vivo (mice, pigs)	Histology, pigmentation, vascularization, wound healing in mice	Tissue model	[354]
Epidermis‐Dermis	Extrusion based bioprinting	Collagen	HFF‐1, HaCaT	Static media and ALI	Histology, markers	Tissue model	[351]
	Extrusion based bioprinting, laser‐assisted bioprinting	Collagen	dermal hFBs, hKTCs	Static media, ALI, in vivo (mice)	Wound healing, mechanics, perfusion	Tissue model	[357]
	Extrusion based bioprinting, bioassembly	Gelatin, Alginate	mice MSCs, KTCs, FBs spheroids	Static media	Histology, markers	Tissue model	[356]
	In Situ inkjet bioprinting	Fibrinogen, Collagen	Autologous FBs, KTCs	in vivo (mice, pigs)	Barrier function, wound healing	Implant	[207]
	Melt Electro Writing	PCL	hFBs, hKTCs	Static media with vitamin C	Mechanical properties and ECM synthesis	Tissue Model	[361]
Epidermis‐Dermis, vascularized	Extrusion based bioprinting	Collagen	hKTCs, hFBs, Pericytes, and ECs	Static media, ALI, in vivo (mice)	Grafting, perfusion	Implant	[355]
Melanoma	Extrusion‐based bioprinting	Agarose, Collagen I	MM A A375, Mel‐1, HUVECs, MSCs and FBs, CSCs	Static media, in vivo (mice)	Vascularization, tumor behavior, gene expression,	Tissue model for cancer	[360]
Melanoma	Extrusion‐based bioprinting	Alginate, Microcellulose	hMel Im, MV3 and HTZ19 (FUCCI), as spheroids	Static media	Mechanical properties, tumor behavior	Tissue model for cancer	[359]
hFBs: human fibroblasts; hKTCs: human keratinocytes; FDPCs: follicle dermal papilla cells; hDMECs: human dermal microvascular endothelial cells; HFF‐1: human fibroblasts; HaCaT: immortal keratinocytes from human skin; MM A A375: malignant melanoma epithelial cells; Mel‐1: melanoma melanocytes; CSCs: cancer stem cells; MV3: human melanoma cell line; Mel Im: human malignant melanoma metastases cells; FUCCI: fluorescence ubiquitination‐based cell cycle indicator; HTZ19: human melanoma cell line.							
Cartilage							
hyaline cartilage (articular)	Extrusion based bioprinting	GelMa, PCL	rat BMSCs; costal chondrocytes	Static media; in vivo (rat)	Morphology (ICC: phalloidin), ECM production, cartilage repair, mechanical testing, gait analysis, gene expression	Implant	[386]
hyaline cartilage (articular)	In‐Situ Extrusion‐based ‐ Coaxial printing with Biopen	GelMa and HAMA	sheep MSCs	in vivo (sheeps)	Cartilage repair assessment, mechanical properties, IHC: collagen, fibrocartilage, hyaline cartilage	Implant	[208]
elastic cartilage (auricular)	Extrusion based bioprinting	dECM‐MA, GelMA PEO, PCL	rabbit auricle chondrocytes	Static media; in vivo (mice)	IHC: collagen, Ki67, alpha‐tubulin, mechanical properties	Implant	[388]
	Extrusion based bioprinting	HA‐SH, PEG‐acryl, PEG‐allyl	human MSCs	Static media	ECM production, gene expression, mechanical properties	Implant	[383]
	Extrusion based bioprinting	dECM, alginate, PCL	Human, porcine BMSCs	Static media (low oxygen)	ECM production, gene expression, mechanical properties	Implant	[374]
	Extrusion based bioprinting	NorHA	MSCs	Static media	ECM production, gene expression, neotissue formation	Implant	[375]
	Extrusion based bioprinting	Nanofibrillated Cellulose, Alginate, HA	iPSCs, irradiated chondrocytes	Static differentiation media	Gene expression, differentiation, ECM production	Implant	[376]
	Extrusion based bioprinting InGel	Fibrinogen, Gelatin	GFP‐HUVECs, hbMSCs	Static media, perfusion	IHC: collagen I and II, ICC: CD31, vascularization	Tissue Model	[449]
	Bioassembly (Kenzan)	Scaffold free	chondrocytes, fibroblasts, MSCs, HUVECs	Static media, perfusion, in vivo (rats)	Mechanical properties, ECM production, IHC: collagen I and IV, CD31, pancytokeratin, vascularization	Implant	[389]
BMSCs: bone marrow mesenchymal stem cells; MSCs: mesenchymal stem cells							
Heart							
Valve, ventricle	Extrusion based printing‐ FRESH	Collagen I	human stem cell derived cardiomyocytes	Static media	Contractility, markers, opening closing	Implant	[450]
Ventricle	Extrusion based bioprinting‐ In‐gel	Collagen I & Hyaluronic acid	hiPSC CMs	Static media	Markers, contractility, calcium flux	Tissue Model	[395]
Heart	Extrusion based bioprinting‐In‐gel	dECM (omentum)	hiPSC CMs, HUVECs	Static media	Markers	Implant	[394]
	Extrusion based bioprinting, MEW	GelMA, collagen, fibrinogen, Matrigel. PCL	hiPSC CMs, fetal cardiac FB, HUVECs	Static media	Contractility, vascularization, markers	Tissue Model	[187]
	Extrusion based bioprinting	Alginate, GelMA	HUVECs, neonatal rate CMs	Perfusion	Mechanical properties, markers, contractility	Tissue Model	[451]
hiPSC CMs: human induced pluripotent stem cells‐derived cardiomyocyte							
Kidney							
Glomerulus	Extrusion based ‐ Coaxial	dECM (kidney), alginate, collagen I, pluronic F 127	Primary human podocytes, glomerular ECs	Perfusion	Barrier function, albumin uptake	Diseased Tissue Model (hyperglycemia induced injury, drug induced injury)	[412]
Glomerulus ‐ Proximal tubule	Extrusion based ‐ Coaxial	dECM (kidney), alginate, pluronic F127	RPTECs, HUVECs	Perfusion, in vivo (mice)	Barrier function, transport	Tissue Model (drug toxicity)	[411]
Proximal tubule	Extrusion based ‐ Coaxial	Alginate, gelatin	Human ciPTECs, (normal and mutated)	Static media	Barrier function, transport, morphology, cystinosis	Disease tissue model	[410]
	Light Based ‐ volumetric	GelMA, SilkMA, pNIPAM	Human ciPTECs	Static media	Viability	Tissue Model	[56]
	Print of sacrificial material	Pluronic F127, gelatin, fibrin	PTEC‐TERT1	Perfusion	Morphology, barrier function, albumin uptake	Tissue Model (for drug toxicity)	[95]
	Print of sacrificial material	Pluronic F127, PEO, gelatin, fibrin	PTEC‐TERT1, GMECs	Perfusion	Barrier Function, transport, hyperglycemia	Diseased Tissue Model	[94]
	Coaxial microfluidic printing	Alginate, fibronectin, gelatin, pectin	HUVECs, pmTEC	Static media	Morphology	Tissue Model	[409]
ciPTECs: human conditionally immortalized proximal tubule epithelial cells; PTEC‐TERT1: human proximal tubular cells immortalized through stable expression of the catalytic subunit of human telomerase reverse transcriptase; GMECs: glomerular microvascular endothelial cells; pmTEC: primary murine tubular epithelial cells; RPTECs: renal proximal tubule epithelial cells.							
Brain and Spinal cord							
Dorsal root ganglia	LIST	Fibrinogen	Primary mouse DRG neurons	Static media	Response to capsaicin (calcium imaging, before and after stimulation)	Tissue Model	[427]
Ventral Midbrain	Extrusion based ‐ Coaxial	Peptides (IVZK and IVFK)	Mouse VM DA neurons, hESCs DA neurons	Static media	Electrophysiological properties(MEA)	Diseased Tissue Model (Parkinson´s disease)	[429]
Cortico‐Striatal circuit	Extrusion based ‐ Coaxial	Fibrinogen‐Thrombin‐Hyaluronic acid	iPSC´s derived cortical glutamatergic neurons, cortical GABAergic neurons, striatal GABAergic neurons and astrocytes	Static media	Electrophysiological properties (Patchclamp, calcium imaging) Astrocytes are responsive to neuronal stimulation (Calcium imaging, glutamate imaging)	Diseased Tissue Model (Alexander disease)	[426]
Cortex	Extrusion based ‐ Coaxial	Gelatin and PEG (GelNB‐PEGdiSH)	Primary rat cortical neurons	Static media	Electrophysiological properties (Calcium imaging, MEA)	Tissue Model	[452]
Spinal cord	Extrusion based bioprinting	Chitosan‐HA‐Matrigel	Rat NSC	Static media	Animal recovery	Implant	[453]
DRG: dorsal root ganglion; VM DA: ventral midbrain dopaminergic; hESCs DA: human embryonic stem cells derived dopaminergic; NSC: neural stem cells.							
Breast							
Breast	Extrusion/Droplet bioprinting	PCL‐decellularized adipose tissue	hASCs, human subcutaneous adipose tissue	In vivo	Breast regeneration	Implant	[440]
Ductal cancer/Solid cancer	Extrusion based In‐gel	Gelatin, HA	MCF7, MDA‐MB‐231 and SKBR3	Static media	Morphology, Genetic expression, Invasion	Disease model (drug screening)	[442]
Breast cancer	FRESH	collagen I, functionalized HA (silk fibrin supp. bath)	21PT, MDA‐MB‐231, HUVEC, mouse tumor organoids, patient‐derived organoids	Static media	Mimicking TME	Disease model	[445]
hASCs: human subcutaneous adipose tissue; 21PT: (breast epithelial) cellosaurus cell line; MDA‐MB‐231: epithelial human breast cancer cell line							

### 3.1 Vasculature

The vasculature, as an organ, delivers oxygen and nutrients to cells in most tissues and transports CO_2_ and metabolic waste. Over time, structural defects such as aneurysms and occlusions or atherosclerosis commonly occur, requiring substitution of the native vessels via bypass surgery. The standard is the use of autologous grafts or artificial polymer tubes.^[^ ^335^, ^336^ ^]^ There is currently interest in alternatives that combine the patient's own cells with artificial support structures.^[^ ^337^ ^]^ Blood vessels present a hierarchical layered structure that increases in complexity proportionally to the channel diameter, spanning from 1 µm for capillaries up to 25 mm for the aorta.^[^ ^338^ ^]^ Smaller capillaries are mostly composed of a single layer of endothelial cells and pericytes for fast exchange with tissues; arterioles or venules add an outer layer of smooth muscle cells and elastic matrix for contraction and pulsatility to generate blood flow; larger arteries or veins further add a third outer layer of fibroblasts and matrix, which is necessary for reinforcement of the walls to withstand the high pressures of blood pumped from the heart.

To produce functional artificial blood vessels, the key features are the multiple cell types and the layered structure. Approaches such as cell seeding on decellularized^[^ ^339^ ^]^ or electrospun scaffolds,^[^ ^340^ ^]^ cell sheet engineering,^[^ ^341^ ^]^ and cylindrical casting,^[^ ^342^, ^343^ ^]^ have been commonly used to fabricate larger tubular structures in the millimeter range. However, these approaches present limitations, such as poor cell density, inhomogeneity, lack of spatial distribution, and precise cell positioning. As an alternative, 3D bioprinting has the potential to produce complex architectures with cellular homogeneity and control over geometry (like bifurcations) and spatial distribution.

Extrusion‐based bioprinting has been successfully used to produce multilayered tubular constructs with inner diameters of different sizes.^[^ ^344^ ^]^ The techniques used range from in‐gel printing^[^ ^173^ ^]^ of tubular structures to coaxial printing^[^ ^345^ ^]^ with sacrificial materials to droplet‐based printing^[^ ^346^ ^]^ or bioassembly.^[^ ^347^ ^]^ Generally, although the constructs have layers with uniform cell seeding and even sprouting and comparable biological functionality to native vessels, they usually lack similar mechanical properties.^[^ ^335^ ^]^

Overall, the mechanical properties that should be considered in biofabricated grafts include the elastic modulus, kinking susceptibility, recovery, suturability and burst pressure.

Some artificial blood vessels have been implanted and perfused in vivo in animals. Nevertheless, bioprinted vessels have potential for the fabrication of tissue models rather than as vascular grafts,^[^ ^348^ ^]^ both for healthy and diseased tissues.^[^ ^336^ ^]^ Given the poor mechanical properties of hydrogels, solutions such as their reinforcement with other materials have been proposed as alternatives to match the J‐shaped stress‒strain curve and typical viscoelastic behavior while also reaching the kPa stress range.^[^ ^335^ ^]^ A promising alternative is to converge conventional bioprinting techniques with MEW or solution electrospinning, since these techniques can be used to easily and precisely tune the mechanics of the constructs without compromising the biocompatible environment that the cells require.^[^ ^349^ ^]^ In a recent approach, MEW was used to produce PCL tubular scaffolds around which cell‐laden hydrogels were precisely polymerized via volumetric bioprinting.^[^ ^188^ ^]^

Despite significant advances in the field, some challenges remain in terms of ensuring proper endothelialization and tight cell distribution over larger vessels, as well as the volumetric production of such vessels during tissue printing. Finally, ensuring perfusion without clogging and flow turbulence remains a challenge.

Macrovascular structures exceeding a diameter of 5 mm have been successful in producing multilayered tubular constructs that resemble the structure of vascular systems. Matching the mechanical properties of native blood vessels remains key for the use of biofabricated vasculature as artificial grafts. This is also of special importance in small‐diameter vessels, where a mismatch of in vivo mechanics could lead to major issues such as flow turbulence or lumen closing. Moreover, bioprinted blood vessels need to mature for long periods under flow conditions to ensure cell maturation and differentiation. The vascularization of tissue constructs is one of the key challenges in deciding whether printing organs is going to be science or fiction.

### 3.2 Skin

The skin is the largest human organ and is the barrier against the external environment; it is necessary for protection against pathogens, sunlight UV, and injury. It regulates temperature and water release and allows for sensing through touch. The skin presents a layered structure: the epidermis is the outermost layer, the dermis is the thicker middle layer, and the hypodermis is an inner fatty layer. Different layers have different functionalities and therefore different compositions. The epidermis is composed of keratinocytes and is the first barrier layer in contact with air, in which keratin is the most common protein. The dermis is mostly composed of fibroblasts in a collagen‐ and elastin‐rich matrix, including nerve endings, hair follicles and sweat glands. The hypodermis is a fatty layer of adipocytes and includes vasculature and innervation.

Skin wounds and defects are common and may result from injury, surgery, or burns, potentially becoming life‐threatening. For larger wounds, skin grafts are necessary, for which autologous skin is taken from elsewhere in the body or dressings are applied, resulting in scarring. To medicate very extensive portions, as in the case of severe burns, tissue replacements are a valid option to explore.^[^ ^350^ ^]^ The key features to mimic when bioprinting skin are hierarchical layering and the air‒liquid interface (ALI) to expose keratinocytes and develop the epidermis; secondary but equally important other aspects to consider are innervation, vascularization and, for more aesthetic reasons, hair and pigmentation.^[^ ^351^ ^]^

To date, different techniques have been used to produce bioprinted skin, mostly extrusion‐based printing, where different inks are used in conjunction with different cell types to make multilayered scaffolds. The most common functionality tests performed in addition to histological analyses involve assessing the transepithelial electrical resistance and tissue mechanical properties.^[^ ^352^ ^]^

The first bioprinted tissues started appearing in 2009, where collagen was used as a matrix and printed alternatively to layers of cells.^[^ ^353^ ^]^ Skin models are remarkably interesting, as they are already employed in many in vivo grafting experiments on animals, where wound healing and pigmentation tests are assessed. One of the most complete bioprinted tissues to date presents the triple layered structure, vascularization, pigmentation, and hair; a coculture of epidermal keratinocytes, melanocytes, dermal fibroblasts, follicle dermal papilla cells, dermal microvascular endothelial cells, and adipocytes was maintained at maturation for 56 days and even engrafted in mice and pigs for wound healing, followed by 90 days. Compared with the control, allograph, and simple hydrogels, the bioprinted construct produced the best results.^[^ ^354^ ^]^

The skin, as the outermost organ, provides the opportunity for in situ bioprinting. The use of an inkjet‐printed porcine model for the management of full‐thickness wounds has already been investigated.^[^ ^207^ ^]^ In an effort to mimic organ complexity, tissues with vasculature^[^ ^355^ ^]^ and sweat glands or hair are printed.^[^ ^356^ ^]^ A French study demonstrated the preclinical validity of bioprinting for skin grafts by printing dermoepidermal substitutes for permanent wound coverage and proving their success by grafting in vivo on mice.^[^ ^357^ ^]^ A Swiss start‐up, Cutiss, is making a groundbreaking contribution to the skin grafting field with its autologous graft hydrogel‐based product. Their hydrogel skin graft with keratinocyte and fibroblast technology was recently used to treat a pediatric patient with massive burn injury,^[^ ^358^ ^]^ providing an example of its applicability but also commercialization and therefore scalability of production through automation.

Skin models can be employed to study skin cancers such as melanoma.^[^ ^359^ ^]^ A sophisticated melanoma model in which three layers containing keratinocytes, fibroblasts and endothelial cells and mesenchymal stem cells in an agarose/collagen type I bioink with cancer stem cells was created. However, only a limited vascular network was achieved.^[^ ^360^ ^]^

The potential industrial applications of skin models and tissues are indeed an interesting aspect to consider, as also highlighted in our survey (Figure 2D); skin models are already in use in the pharmaceutical and cosmetic industries for testing the chemical composition of products. A pioneer in the industry of development of skin models is certainly L'Oréal, who – through its side company Episkin – develops commercially available skin in vitro models and more recently showed remarkable results with a layered MEW scaffold to support tissue culture for full differentiation and the production of newly synthesized ECM in 18 days.^[^ ^361^ ^]^

Different materials and convergence of techniques make it possible to recapitulate (i) the mechanical properties of native skin (skin stiffness range is 0.05–2 MPa),^[^ ^362^ ^]^ as well as (ii) the layered structure, for which additive manufacturing is highly suitable.

Considering the abovementioned advances, bioprinting hierarchical skin tissues is closer to science than to fiction. The reality of bioprinting skin is most likely the closest to being employed in a clinical setting, pending regulatory approval, of course. Different in vivo studies have proven the efficacy of these methods for wound healing and burn closure, even when they are employed in preliminary clinical trials.^[^ ^363^ ^]^ In addition to medical applications, skin tissue models are already being employed in industry for testing chemicals and cosmetics, making the concept of bioprinted skin a reality.

### 3.3 Cartilage

Cartilage is a specialized connective tissue characterized by low amounts of blood and lymphatic vessels, innervation, and chondrocytes, leading to a limited capacity for self‐healing. Its specialized ECM contains collagen, elastin fibers, proteoglycans, glycosaminoglycans, and water.^[^ ^364^ ^]^ Three different types of cartilage exist depending on their anatomical location and differ in their composition, structure, and therefore specific functions.^[^ ^364^, ^365^, ^366^, ^367^, ^368^, ^369^, ^370^ ^]^ First, hyaline cartilage is the most abundant type found in articulating surfaces, granting a low frictional coefficient and shock absorption as well as providing structural support (nose and ribcage).^[^ ^364^, ^365^, ^366^, ^367^, ^368^, ^369^, ^370^ ^]^ It is composed of different zones, which vary in their ECM composition and in their fiber organization in an arched way to withstand different forces.^[^ ^364^ ^]^ Second, elastic cartilage is characterized by a high content of elastin fibers. It is stiff but flexible enough to provide structural support.^[^ ^368^ ^]^ The third type, fibrocartilage, is characterized by parallel collagen bundles and is very dense to withstand high compression and shear forces^[^ ^368^, ^369^ ^]^ in the meniscus or annulus fibrosus in the intervertebral disk.^[^ ^370^ ^]^ The challenge for bioprinting cartilage lies in recreating these complex and diverse mechanical porperties. This is particularly crucial for the zone representing maturation; it must link both ECM production or degradation due to the incorporated cells and provide mechanical stimuli, which can be simulated during maturation.^[^ ^371^, ^372^ ^]^

For bioprinting cartilage, mesenchymal stem cells (MSCs) and iPSCs,^[^ ^373^, ^374^, ^375^, ^376^ ^]^ as well as chondrocytes,^[^ ^365^, ^377^, ^378^ ^]^ are typically used in extrusion‐based printing,^[^ ^373^ ^]^ or inkjet printing.^[^ ^365^, ^378^, ^379^ ^]^ The selection of bioinks is strongly influenced by the complexity of cartilage mechanics. Natural polymers, such as HA^[^ ^380^ ^]^ or gelatin,^[^ ^381^ ^]^ often fail to provide the mechanical properties needed because of their low stability, rarely achieving native compression moduli.^[^ ^99^, ^371^, ^382^ ^]^ Synthetic polymers can provide mechanical resistance against loading. Current biofabrication approaches often combine natural and synthetic polymers by crosslinking and can even tether growth factors to the bioink, leading to supported tissue development.^[^ ^134^, ^383^ ^]^

To regenerate damaged cartilage, hydrogels alone are not enough to withstand mechanical forces; therefore, studies often combine synthetic biomaterial reinforcement fibers with bioinks.^[^ ^371^, ^384^, ^385^ ^]^ One approach uses a reinforced GelMA ink containing bone marrow‐derived MSCs as well as costal chondrocytes to improve articular cartilage regeneration in rats.^[^ ^386^ ^]^ Another study aimed to repair chondral defects in a preclinical large animal model via in situ printing of a HA‐GelMA scaffold with a handheld 3D coaxial extrusion‐based biopen, leading to early signs of cartilage regeneration.^[^ ^208^ ^]^ After cultivation, a comparable Young's modulus, equilibrium modulus, and maximum stress at 30% deformation of the host cartilage were achieved. However, it remains uncertain whether the scaffold remained in place due to an insufficient amount of adhesive.^[^ ^208^ ^]^

The adhesive property of cartilage was addressed by bioprinting different zones of hyaline cartilage with three hydrogels. This approach fully exploits the advantages of 3D bioprinting via the precise layer‐by‐layer fabrication of a lubrication top layer, a mechanical support middle layer, and an adhesive bottom layer. This model, with low friction, high strength, and adhesion to underlying tissue, exhibited good mechanical performance, effective friction reduction, and short‐term biocompatibility as well as stability.^[^ ^387^ ^]^

Another application for bioprinting is replacement, e.g., auricles, to help patients suffering from complete loss of the outer ear or parts of it. Auricles are biofabricated via the use of a modified acellular cartilage matrix bioactive bioink reinforced with PCL fibers via multinozzle extrusion‐based bioprinting. The printed scaffold first matured in vitro and was subsequently implanted into mice, where it continued to mature. This led to a successfully fabricated auricular equivalent with sufficient mechanical strength and well‐defined shapes. During mechanical characterization, the reinforced scaffold exhibited greater stiffness and increased compression loss than did native tissue, but it also demonstrated better shape fidelity than the scaffolds without PCL.^[^ ^388^ ^]^ Great success has also been achieved in the attempt to biofabricate trachea‐like, scaffold‐free structures for the replacement of the trachea. Spheroids were generated and bioprinted as trachea‐like models via the Kenzan method, matured in a bioreactor, and implanted in rats. Considering the mechanical aspects of the bioprinted models, the ultimate tensile strength (UTS) was significantly lower, and the force at failure was comparable, indicating an overall lower mechanical strength than that of native tissue.^[^ ^389^ ^]^

Promising approaches, such as the use of multiple hydrogels to mimic zonal structures of hyaline cartilage^[^ ^387^ ^],^ and stepwise strategies, such as replacing damaged cartilage with autologous cartilage harvested from less load‐bearing regions^[^ ^390^ ^],^ are currently under investigation. Nevertheless, to truly move cartilage bioprinting from fiction to science, we need to address key bottlenecks such as mechanical complexity. The combination of different bioinks to achieve this range of mechanical properties while maintaining printability is essential, as is a stronger focus on tissue maturation, particularly considering the interplay between dynamic mechanical loading, cell metabolism, and ECM production. The state of the art is defined by partial defect repair, in vitro disease models, and short‐term in vivo cartilage reconstruction in animal models. However, the prospect of fully restoring functional cartilage is no longer fiction but rather supported by tangible scientific progress.

### 3.4 Heart

While only about the size of a clenched fist, the human heart pumps approximately 220 million liters of blood through our body during our lives, accumulating in approximately 88 Olympic swimming pools. Our heart starts beating as early as 3 weeks after conception and continues to beat approximately 100000 times per day. Nevertheless, the heart's ability to sustain rhythmic and coordinated contractions can deteriorate, leading to severe systemic dysfunctions. Cardiovascular disease is the leading cause of death worldwide, and with the number of cases increasing every year, the need for nonxenogenic pharmaceutical screening platforms as well as whole‐organ replacement is growing. With the ultimate goal of creating a fully functional human heart, cardiac tissue engineering faces the challenge of replicating the layered structure of the heart with the endo‐, myo‐, and epicardium while creating a functional synergy between the atria, ventricles and valves. The heart is further characterized by multiple cell types, a dense microvasculature, an anisotropic structure and genetically highly diverse subcategories of cells, such as the division of cardiomyocytes into chamber‐specific as well as compact and trabecular subtypes.^[^ ^391^, ^392^, ^393^ ^]^

In line with this demanding challenge, there appears to be a mismatch between complex hierarchical and advanced biological requirements in most cardiac tissue engineering strategies. The attempt at structural replication of a heart with the 3D printed model of a four‐chambered heart, including vessel‐like structures, did not result in spontaneous and rhythmic contractions.^[^ ^394^ ^]^ Nevertheless, this model includes multiple cell types and displays a relatively high degree of architectural complexity, highlighting progress in recapitulating the cellular diversity of the heart. In contrast, a single ventricle‐like model that shows spontaneous contractions for 100 days and responds to adrenergic stimulation could be directly bioprinted.^[^ ^395^ ^]^ This finding demonstrates that cardiomyocytes within a large‐scale 3D printed construct are not only able to initiate autonomous contractions but also able to maintain them over long periods of time. While neither of the two model approaches the human heart in terms of functionality or complexity, they still provide encouraging signs of progress. While many approaches in the past have employed rodent cardiomyocytes, recent publications have focused on the use of human iPSCs, as they are nonxenogenic, provide the option to modify them according to specific pathologies and can be generated in a patient‐specific manner. However, stem cell‐derived cardiomyocytes are usually immature, and the contractile forces generated by such cell assemblies remain below those required to match the performance of an adult human heart.^[^ ^393^ ^]^ The hallmarks of cardiomyocyte maturity are adult‐like gene expression, a sarcomere length of 2.2 µm, the presence of transverse tubules, oxidative metabolism and mature calcium handling, as expressed by positive force‐frequency handling.^[^ ^396^ ^]^ Accordingly, techniques to enhance the maturation of mainly casted engineered myocardial tissues have been investigated,^[^ ^397^, ^398^ ^]^ which may be translated to 3D printed models in the future. In addition to cardiomyocytes, other cell types commonly used include endothelial cells, fibroblasts and immune cells.^[^ ^399^ ^]^ A variety of techniques have been implemented, ranging from classical extrusion‐based bioprinting to in‐gel printing or sacrificial writing into functional tissues, a 3D printing method in which cardiac organoids are used to achieve increased cell densities.^[^ ^174^ ^]^ Typical strategies employ natural materials such as fibrin and collagen, which can be supplemented with synthetic components such as PLA or PCL to achieve increased levels of cardiomyocyte maturation due to improved mechanical properties.^[^ ^391^ ^]^ In addition, the use of fibers for better cell alignment^[^ ^399^ ^]^ or engineered proteins such as silk fibroin to tailor cellular adhesion and degradation kinetics is highly promising.^[^ ^400^ ^]^ These biomaterials provide important steps forward, but they cannot yet recreate the full mechanical and electrical integration required for whole‐organ function.

Apart from the full reconstruction of the human heart or parts thereof, several other areas are currently being explored in the field of cardiac tissue engineering, such as cardiac patches,^[^ ^401^ ^]^ injectables^[^ ^402^ ^]^ and heart‐on‐a‐chip platforms.^[^ ^403^ ^]^ While aiming at bridging infarct‐impaired tissue, cardiac patches often suffer from several shortcomings, such as insufficient conduction velocity, weak mechanical properties, missing or insufficient vasculature and inadequate cell alignment.^[^ ^399^ ^]^ Heart‐on‐a‐chip platforms contrast and are not intended for direct implantation in human recipients but serve to advance cardiovascular research and personalized therapy development while overcoming the limitations of animal‐based models.

The abovementioned results suggest that although the goal of printing a fully functional heart may remain fiction for now, the possibility cannot be entirely dismissed, and incremental advances continue to offer grounds for cautious optimism. The science of creating contractile cardiac tissue with measurable force output is advancing, but the fiction remains the ability to generate cells that reproduce the full power and coordinated behavior of an adult human myocardium. Given the variety of strategies and components of the heart that have been investigated, a future approach is likely to benefit from combining the mentioned research to create functional synergy. However, despite some encouraging steps in the right direction having been made, no research has yet demonstrated the full structural complexity or comprehensive functionality of even a fetal heart. Accordingly, it remains clear where science currently stands—patches, chips, partial tissues, and maturing models—and where fiction begins: the prospect of a fully printed, functional human heart.

### 3.5 Kidney

Healthy kidneys filter approximately 1600 liters of blood daily, producing 180 liters of primary urine. However, owing to the reabsorption ability of the kidneys’ tubular compartment, only 1 to 2 liters of urine are excreted per day. The remaining 99% is transported back into the bloodstream, where it regulates fluid and electrolyte homeostasis in the body and blood pressure.^[^ ^404^, ^405^, ^406^ ^]^ Kidneys are highly intricate organs composed of various compartments, multilayers, and several cell types, making their biofabrication extremely challenging. Furthermore, until now, there have been no bioprinting techniques that achieve the required resolution for renal structures. However, there is increasing public interest in advanced kidney models and organ replacement due to the increasing incidence of kidney disease, the irreversible nature of chronic kidney disease, the aging population, and limited treatment options.

A bioartificial kidney has yet to be achieved, but some promising alternative approaches to conservative replacement therapy have been proposed. For example, scaffolds with postseeded progenitor cells have been shown to support kidney tissue regeneration in a chronic kidney disease mouse model,^[^ ^407^ ^]^ whereas decellularized kidney scaffolds guided cell attachment and growth.^[^ ^12^, ^408^ ^]^ More commonly, biofabrication efforts are focused separately on printing different nephron components, from the glomerulus to tubular compartments. Over the past decade, several studies have focused on the biofabrication of the proximal convoluted tubule as a segment, which is crucial for reabsorption and is a significant injury target in the progression of acute renal failure. One innovative technique involves the use of printed sacrificial ink embedded in an engineered ECM to create a perfusable 3D structure that mimics the convoluted tubule^[^ ^95^ ^]^ and vascular channel.^[^ ^94^ ^]^ Proximal tubule epithelial cells (PTECs) seeded into the tubular channel demonstrated improved epithelial morphology (cell polarity and primary cilia formation) and barrier function. Under hyperglycemic conditions mimicking diabetic nephropathy, epithelium‒endothelium crosstalk was revealed, highlighting the importance of complex models for disease studies. A typical strategy to produce double‐layered tubes is coaxial printing. Therefore, this technique was applied to create a double‐layered straight filament carrying human umbilical vein endothelial cells (HUVECs) in the sheath and tubular epithelial cells in the core layer.^[^ ^409^ ^]^ After the maturation period, the core part remodeled, and a hollow channel formed.

Recently, the convoluted architecture and small diameter of the proximal tubule were addressed.^[^ ^410^ ^]^ The printed microfibers contain a helical perfusable microchannel in a custom‐designed microfluidic chip. In this system, the barrier formation, cell polarization, and transporter activity of postseeded PTECs and the cystinosis knockout cell line were analyzed. An alternative technique for two parallel channels representing the proximal tubule and blood vessel compartments was introduced.^[^ ^411^ ^]^ Tubes were fabricated with hybrid bioinks containing PTECs and HUVECs via coaxial printing. The system demonstrated specific albumin reabsorption and transport between the two compartments. Finally, volumetric printing followed by thermal treatment was proposed as an innovative method for fabricating the proximal tubule compartment.^[^ ^56^ ^]^ By using the shrinking properties of GelMA and silkMA in combination with temperature‐responsive pNIPAM, the authors successfully created complex hollow tubular structures. Notably, the GelMA‐pNIPAM combination resulted in the smallest channel diameter achieved to date, along with enhanced cytocompatibility for PTECs.^[^ ^56^ ^]^

Despite the architectural challenges of a glomerular filtration barrier consisting of three layers (fenestrated endothelial cells, the glomerular basement membrane, and podocytes forming slit diaphragms), the first printed model for the glomerulus has been reported.^[^ ^412^ ^]^ Using kidney‐dECM bioink and coaxial bioprinting, the authors achieved a perfusable microvessel‐on‐a‐chip consisting of glomerular endothelial cell and podocyte layers, demonstrating a selective permeability capacity and response to nephrotoxic drugs and high glucose conditions.

In addition to the studies mentioned above, the high variability of other approaches, such as on‐chip systems, artificial membranes, and bioreactors, are actively applied in kidney research, aiming to fulfill a greater degree of model maturation.^[^ ^413^ ^]^

Kidneys are composed of a multitude of compartments at a variety of scales, all nested and connected to each other, making the architecture of an organ particularly complex. While it has been possible to recapitulate singular aspects of the kidney, contemporary printing techniques are still not sufficient to print to scale parts. Therefore, a strategy that is more likely to be successful would be to exploit developmental mechanisms to grow interconnecting sections from cellular building blocks. Similarly, cells may recreate the miniscule architecture by engineering their own environment while providing macro spatial organization, perfusability and the correct spatial cues.

### 3.6 Brain and Spinal Cord

The brain operates as the main processing hub in the human body, where the central nervous system (CNS) and peripheral nervous system (PNS) serve to gather and relay stimuli.^[^ ^414^ ^]^ The information from the internal state of the body and our surrounding environment is interpreted to respond accordingly.^[^ ^415^ ^]^ Different cell types and tissues hierarchically structure a rigorously balanced system. Both CNS and PNS neurons constitute the main functional unit, whereas glial cells provide support, which is fundamental for neurons to function and survive.^[^ ^416^ ^]^ At a glance, seemingly random connections all through the CNS closely communicate;^[^ ^417^ ^]^ however, the brain circuitry topography is carefully curated to generate efficient and robust neural networks.^[^ ^418^ ^]^

Mechanical, chemical and topographical cues ensure that during development and as we age, the whole nervous system continues to function^[^ ^284^ ^],^ guiding a myriad of neuronal processes from development to maturation, such as axonal guidance and cortex folding.^[^ ^285^ ^]^ The ECM of the brain is primarily composed of soft hyaluronic acid, and low levels of collagen IV are present only in the basement membrane of the brain ECM. Moreover, proteins such as laminin, fibronectin and tenascin are key for the development and function of neurons and glial cells.^[^ ^419^ ^]^ Changes in the ECM are present in various neurodegenerative diseases, e.g., Alzheimer's disease,^[^ ^420^ ^]^ Parkinson's disease,^[^ ^420^ ^]^ and schizophrenia.^[^ ^419^ ^]^ Neurons can achieve very complex brain functions only when all mechanical cues, ECM organization and cell type requirements are met; therefore, for bioprinting brain tissue, several conditions need to be considered. Compliance with chemical and mechanical compositions is necessary. Specifically, brain tissue is ultrasoft with stiffnesses in the range of 100–2000 Pa, depending on the developmental stage. Moreover, spatial resolution differs for different neuronal circuits that define specific regions, e.g., the cortex, basal ganglion, and thalamus. It is key to ensure long‐term maturation processes that support the functional development of heterocellular neuronal networks. In this manner, one can secure the basic requirements for the generation of in vitro neuronal tissue.

The current state of the field highlights two specific areas of development that show the clinical relevance and potential of bioprinted constructs: injury repair and disease models. The use of bioprinted constructs has been shown to be relevant for injury repair, where the implantation of 3D printed stem cell hydrogels promotes and improves recovery in mouse models after traumatic brain injury and spinal cord injury.^[^ ^421^ ^]^

Several in vitro models that construct the environment of neurons have been developed using different bioinks and printing approaches.^[^ ^422^, ^423^, ^424^, ^425^ ^]^ Chitosan has been used for long‐term culture of 3D neuron‒astrocyte biofabricates.^[^ ^39^ ^]^ Moreover, the automation of bioprinting approaches has allowed high‐throughput studies, such as those with 2D in vitro assays, to be obtained. These studies are fundamental for the development of new therapeutics. Considering the CNS, an extrusion bioprinted construct with iPSC‐derived neuronal and glial cells was developed, resulting in different cell types that are structurally and functionally mature. Additionally, a bioprinted layered construct with cortical and striatal neurons generated a cortical‐striatal functionally active neuronal network,^[^ ^426^ ^]^ taking bioconstructs one step closer to obtaining 3D printed neural circuits. PNS mimicking cultures of dorsal root ganglia generated via laser‐induced side transfer bioprinting displayed mature neurons that had proper electrophysiological properties and responded to capsaicin.^[^ ^427^ ^]^

Neurological disorders are the leading cause of mental and physical disabilities worldwide, with 15% of the world's population affected by one of these diseases. With neurodegenerative disease prevalence expected to double in the following decades,^[^ ^428^ ^]^ modeling these diseases to better understand them and finding cures is something that biofabrication is helping with. Bioprinting has been employed in Parkinson's disease models; one study used 3D bioprinted dopaminergic neurons in a biomimetic peptide scaffold, whereas another study bioprinted human dopaminergic neurons with a coculture of human astrocytes and differentiated monocytes in an ECM‐derived bioink doped with electroconductive nanostructures to model neuroinflammatory responses.^[^ ^429^, ^430^ ^]^

The bioprinting of functional neuronal tissue is still fiction. Nonetheless, the current state of functional bioprinted neuronal circuit models has achieved a great level of complexity and functionality. It has become possible to produce in vitro neuronal networks capable of generating spontaneous, robust and constant activity.^[^ ^431^, ^432^, ^433^ ^]^ Moreover, these bioprinted models have been shown to recapitulate disease mechanisms reported in vivo, highlighting their viability for investigating and unraveling the mechanisms of neurodegenerative and neurological diseases.^[^ ^434^ ^]^

### 3.7 Breast

The human breast is a complex structure composed of epithelial, adipose, connective, and glandular tissue. While present in both sexes, breasts become more prominent in females after puberty because of the effects of estrogen and progesterone, which stimulate the accumulation of adipose tissue and the maturation of mammary glands. These glands, consisting of ducts and secretory lobules, play a critical role in lactation. The female breast remains highly sensitive to hormonal fluctuations throughout the menstrual cycle, pregnancy, and menopause, reflecting its dynamic nature. The underlying ECM, which is composed primarily of collagen I, collagen IV, and laminin provides mechanical support, ensures structural integrity, and facilitates essential mechanical and biochemical signals fundamental to the development, maintenance, and regeneration of adipose tissue.^[^ ^435^ ^]^ The main challenge in bioprinting functional breast tissue involves engineering a scaffold that simultaneously promotes adipocyte maturation by providing a suitably soft matrix while ensuring sufficient mechanical integrity to maintain structural support.^[^ ^435^, ^436^ ^]^

Breast cancer ranks as the most frequently diagnosed cancer globally and is the second most common cause of cancer‐related death in women.^[^ ^437^ ^]^ Since, for nonmetastatic breast cancer, the standard therapy remains surgical resection,^[^ ^438^, ^439^ ^]^ rates of breast reconstruction have increased, increasing the demand for new implantable materials that offer better safety, longevity and cosmetic outcomes. While breast tissue regeneration via 3D bioprinting is still at its early developmental stages as a potential alternative to conventional implants and autologous flaps, some studies have shown promising results. For example, constructs composed of human adipose‐derived stem cells within a decellularized adipose tissue bioink printed on a PCL scaffold demonstrated adipose tissue formation, angiogenesis, and host tissue infiltration after implantation in mice.^[^ ^440^ ^]^ Another approach relies on cell seeding on 3D‐printed breast‐shaped PCL scaffolds, which, after implantation in mice and subsequent fat injection, results in constructs with stiffness values (2.9±0.6 kPa) comparable to those of native adipose tissue, along with vascularization and an adipose‐like morphology. However, inflammatory responses, including capsule formation and immune cell infiltration, were also observed.^[^ ^441^ ^]^ Similarly, a start‐up developed 3D printing and lipofilling methods, which are currently in the preclinical stage. The Wyss Institute's Reconstruct project (Harvard University), on the other hand, focuses on bioprinting patient specific, vascularized adipose tissues for breast reconstruction.

Nevertheless, the main bioprinting applications regarding breast tissue currently focus on recapitulating breast cancer in vitro to investigate tumor–stroma interactions and evaluate therapeutic responses in a more representative microenvironment. For example, breast cancer models accurately representing the different clinical stages of breast cancer were bioprinted using a collagen/alginate/HA composite and three breast cancer cell lines.^[^ ^442^ ^]^ However, the importance of reciprocal interactions between cancer cells and their surrounding stromal cells was not considered. Recently, such interactions between adipose‐derived stem cell spheroids or adipose spheroids and single breast cancer cells were investigated via disk‐ring geometry, which revealed that stromal cells increased key migration parameters of epithelial adenocarcinoma cells.^[^ ^443^ ^]^ Similar geometries have been used to study the effects of adipose‐derived stromal cells on drug resistance in an attempt to mimic the in vivo conditions of the early stage of breast cancer. These findings suggest that the presence of adipose‐derived stromal cells indeed contributes to chemotherapy resistance.^[^ ^444^ ^]^ All the abovementioned models were generated via classical extrusion‐based bioprinting. Recently, embedded bioprinting^[^ ^173^ ^]^ has been increasingly used to create sophisticated tumor‐stroma models because it allows the printing of softer biopolymers. For example, using a collagen I and HA‐pNIPAM bioink in a silk fibrin support bath, mouse tumor‐derived organoids were printed and were able to maintain cancer and stromal cell phenotypes.^[^ ^445^ ^]^ Similarly, breast cancer spheroids bioprinted into a support bath composed of adipose tissue‐derived cells and ECM have been shown to respond appropriately to the chemotherapeutic agent doxorubicin.^[^ ^446^ ^]^ Although bioprinting offers great potential for patient‐specific drug testing, its broader impact will depend on the integration of more complex, multicellular environments, dynamic perfusion systems, and systematic validation across large patient cohorts. Once these challenges are addressed, bioprinted cancer models could serve as platforms for personalized medicine, both to better predict individual patient drug responses and to aid in the discovery of novel therapeutics.

The vision of bioprinted breast reconstruction is compelling; however, to the best of our knowledge, no clinically applicable 3D bioprinted breast reconstruction is possible, and current research addresses challenges related to vascularization, immune compatibility, and long‐term tissue stability. However, bioprinting breast tissue already plays a central role in advancing in vitro tumor modeling for cancer research.

## 4 Perspective: Opportunities and Challenges in Biofabrication

### 4.1 A Young Discipline with Rapid Momentum

Bioprinting and biofabrication are remarkably young disciplines, barely two decades old, yet they have already evolved from visionary ideas into powerful tools that are reshaping tissue engineering. What once seemed unrealistic has now become experimentally achievable, driven by the convergence of precise analytical methods, the rapid expansion of biological and biochemical understanding, and the emergence of novel biomaterials and advanced processing technologies, including hydrogels, composite matrices, nanostructured bioinks, and stimuli‐responsive polymers that mimic the ECM and enable cell instruction. The pace of progress is impressive, reflecting both the creativity and interdisciplinarity that define this field. Although not all links within this complex network of disciplines are fully established, we are clearly in an active and accelerating phase of development. If the community continues to integrate expertise across domains, further breakthroughs are inevitable.

The opportunities ahead lie not in any single breakthrough but in the ability to connect the many rapidly evolving facets of biofabrication. Advances in technology, data‐driven design, and material intelligence must now converge into an integrated framework that unites precision engineering with biological maturation — a challenge that will define the next phase of the field.

### 4.2 Technological Convergence and Interdisciplinary Integration

The biofabrication of tissues and organs represents one of the most ambitious frontiers in modern biomedicine. It unites previously distinct disciplines—biology, medicine, materials science, chemistry, and engineering—around the shared goal of recreating living, functional human tissues. Technological convergence and interdisciplinary collaboration are therefore fundamental to advance organ and tissue model fabrication. The field arises from the integration of cell biology, biomaterials science, and mechanical engineering,^[^ ^454^ ^]^ which requires close cooperation among multidisciplinary teams to overcome persistent barriers.^[^ ^455^ ^]^

Progress has been enabled by the integration of multiple manufacturing technologies into unified biofabrication platforms, which outperform single‐component systems in functionality and versatility.^[^ ^456^ ^]^ Modern extrusion, inkjet, laser‐assisted, stereolithographic, and volumetric bioprinting methods—often combined in hybrid or multimodal systems—enable cell and biomaterial deposition with micron‐scale precision. Parallel advances in microfluidics, imaging, omics, and rheological characterization have deepened our understanding of how structure and composition define tissue function. The convergence of scaffold‐based 3D bioprinting with scaffold‐free bioassembly offers new opportunities to fabricate complex, multicellular constructs.^[^ ^457^ ^]^ Likewise, integrating organoid technologies with biofabrication helps overcome current limits in terms of organoid size and physiological realism.^[^ ^458^ ^]^ Core design principles must now align developmental, anatomical, and physiological insights with enabling technologies such as adaptive biomaterials and programmable fabrication platforms.^[^ ^459^ ^]^

This multitechnology, knowledge‐driven approach—supported by automation, standardization, and scalability across laboratories and further enhanced through artificial intelligence—represents the foundation of next‐generation tissue manufacturing.^[^ ^460^, ^461^ ^]^ The resulting feedback loop between fabrication and analysis, where material design, biological response, and process optimization continuously inform one another, is expected to accelerate both discovery and clinical translation in the coming decade.

### 4.3 Artificial Intelligence and Computational Design

Artificial intelligence (AI) is transforming biofabrication and tissue engineering by addressing the complex interdisciplinary challenges of organ and tissue model development. It enables the integration of large, heterogeneous datasets spanning biology, materials science, and engineering.^[^ ^462^ ^]^ In 3D bioprinting, AI‐driven and machine learning approaches improve medical image reconstruction, bioink selection, and process control, allowing the precise deposition of living cells and biomaterials for functional tissue fabrication.^[^ ^463^ ^]^

AI now supports all stages of additive manufacturing—from design and parameter optimization to real‐time monitoring and postprocessing—enhancing both structural fidelity and reproducibility.^[^ ^464^ ^]^ Machine learning methods such as 3D convolutional neural networks can also predict the mechanical properties of tissue scaffolds on the basis of virtual tomography data.^[^ ^465^ ^]^ In addition to process control, AI contributes to predictive modeling of vascularization, organoid evolution, and tissue maturation, linking imaging and computer‐aided design to enable patient‐specific construct generation. By translating biological complexity into actionable design parameters, AI enhances efficiency, scalability, and data‐driven decision‐making in biofabrication.

AI is expected to play a catalytic role in integrating computational design, smart materials, and bioprinting technologies. These AI‐driven strategies are accelerating the evolution of regenerative medicine—advancing personalized therapies, organ transplantation, and the replacement of animal experiments.^[^ ^462^ ^]^

### 4.4 Predictive Modeling and Digital Simulation

Beyond the integration of fabrication technologies, the next leap forward will come from predictive modeling and digital simulation, enabling in silico experimentation and digital twins to forecast how formulations, printing parameters, and maturation cues influence construct performance. These computational approaches address both the complexity and cost challenges inherent in tissue engineering by translating biological processes into mathematical models that can be iteratively refined before expensive in vitro experiments. They support process modeling, design, and optimization while accounting for biological complexity.^[^ ^466^ ^]^ Cellular particle dynamics simulations can predict postprinting self‐assembly behavior, enabling efficient biofabrication of tubular organ structures without complex control experiments.^[^ ^467^ ^]^ Computational fluid dynamics (CFD) models help evaluate bioink printability and optimize printing parameters, thereby reducing development time and cost.^[^ ^468^ ^]^ Machine learning–aided databases are now used to predict optimal printing parameters and organ functionality metrics by analyzing large collections of research data.^[^ ^469^ ^]^ Computational models also support postfabrication prediction of tissue evolution and functional performance,^[^ ^470^ ^]^ whereas numerical simulations allow forecasting of filament behavior during direct ink writing and other extrusion‐based processes.^[^ ^471^ ^]^

This shift toward computationally assisted design will reduce reliance on trial‐and‐error experimentation and move the field toward rational, data‐guided process design. In this sense, precision must extend beyond the development of materials and processes to the design of research itself toward hypothesis‐driven, predictive experimentation.

### 4.5 From Printing to Maturation

Even the most advanced bioprinters cannot yet create fully functional tissues or organs. Current approaches generate precursor constructs, which are architecturally defined templates that require maturation to achieve physiological function. These precursors embody a shift in tissue engineering, drawing on developmental biology to guide organ formation from engineered rudiments rather than printing fully mature tissues.^[^ ^472^, ^473^ ^]^

Tissue spheroids are often used as building blocks that undergo fusion and self‐assembly, mimicking morphogenetic processes during embryogenesis.^[^ ^474^, ^475^ ^]^ This developmental engineering concept enables spatiotemporal control over cell–cell and cell–matrix interactions, supporting the formation of complex structures with vascularization and tissue‐specific functionality.^[^ ^455^, ^461^ ^]^ Integrating these principles into 3D bioprinting allows the regulation of signaling pathways and the creation of tissue‐like architectures through guided self‐organization.^[^ ^476^ ^]^ Maturation depends on orchestrated cellular interactions supported by dynamic perfusion, controlled degradation, and biochemical signaling. Bioreactors provide the necessary microenvironment with physical and biochemical cues that drive cell proliferation, differentiation, and functional development.^[^ ^477^ ^]^ Advanced systems capable of delivering oxygen, nutrients, and mechanical stimuli extend biofabrication beyond printing, emphasizing it as a continuous process where cells and materials coevolve.

This approach closely mirrors natural developmental processes, offering a biological blueprint for in vitro tissue formation. Here, the bioreactor functions not only as a culture vessel but also as a controlled environment for self‐organization and maturation, ultimately guiding the transition from printed constructs to living, functional tissue.

### 4.6 Material Intelligence and 4D Biofabrication

Material intelligence and 4D biofabrication represent a paradigm shift in tissue engineering, incorporating time as the fourth dimension through stimuli‐responsive smart materials that dynamically evolve after fabrication.^[^ ^478^, ^479^ ^]^ Unlike static 3D bioprinting, 4D approaches employ programmable biomaterials that can change their shape, stiffness, or functionality in response to external or physiological stimuli, more closely mimicking native tissue dynamics.^[^ ^480^, ^481^ ^]^

The success of tissue maturation relies on such active materials, which transition from passive scaffolds to instructive matrices capable of guiding cellular behavior. By encoding degradation kinetics, stiffness gradients, and bioactive ligand patterns, they provide spatially and temporally defined cues that drive differentiation and tissue organization. Smart hydrogels, stimuli‐responsive polymers, and hybrid fiber–hydrogel composites are particularly promising, as they adapt dynamically to cellular remodeling and environmental signals.^[^ ^482^, ^483^ ^]^ This material diversity spans natural, synthetic, and ECM‐mimicking hydrogels; designer matrices; and smart composite bioinks capable of time‐dependent adaptation, collectively known as 4D bioprinting. Naturally, derived biomaterials are particularly advantageous for creating cell‐rich constructs with shape‐memory and morphing features.^[^ ^484^ ^]^ Integration with artificial intelligence further enhances design capabilities and manufacturing precision, enabling data‐driven optimization of material composition and functional transformation.^[^ ^485^ ^]^

Taken together, such material intelligence offers a pathway to bridge engineered constructs with living, self‐organizing tissue, providing the foundation for next‐generation regenerative medicine.

### 4.7 Outlook

In the future, interdisciplinary collaboration will remain the key driver of innovation. Engineers must adopt biological thinking, whereas biologists must engage with the principles of design and manufacturing. Only through such cross‐pollination can we align the mechanical precision of fabrication with the adaptability inherent to living systems. The next generation of biofabrication platforms will not merely replicate anatomy; rather, they will orchestrate the dynamic processes that define life. The challenge is formidable, but so too is the opportunity: to move from constructing form to cultivating function. Our accompanying survey of both experts and nonexperts points in the same direction. Biofabrication is widely viewed as societally meaningful and ethically sound, with near‐term potential for tissue models, tissue parts, and testing, whereas experts remain more cautious about timelines. This broad confidence reinforces the case for coordinated progress.

The next decade will be decisive for translating biofabrication from an experimental framework into clinical and industrial reality. Progress will rely on predictive, data‐guided design, intelligent materials, and a deeper understanding of developmental processes that enable controlled maturation and functional integration of engineered constructs.

## 5 Epilogue—Students’ Perspective

This epilogue reflects the viewpoint of doctoral researchers who work daily at the interface of materials, engineering, and cell biology. It complements the preceding perspective by focusing on what enables steady progress at the bench: robust protocols, transparent reporting, and practical training that spans disciplines. This epilogue discusses scalability, maturation, and functional readouts and outlines practical steps to reduce variability, shorten iteration, and align research with clinical and industrial translation. The emphasis is on turning promising concepts into routine, reproducible practices in typical laboratory settings.

### 5.1 Practical challenges

Practical issues include the fact that synthesizing and modifying materials can take many hours, with small yields and batch‐to‐batch variations, especially for natural polymer derivatives; additionally, bioinks have a limited shelf life when reconstituted as hydrogels. During extended preparation and printing times, the material could incur changes in properties, reducing reproducibility even within the same experiment, or cells may perish if exposed to harsh printing conditions too long. A common conceptual and technical challenge for materials is balancing printability and biocompatibility.

Commercial printers are user friendly and have practical, minimalistic interfaces but often limit control or feedback over parameters, making them less applicable for advanced uses needed for bioprinting functional organs. Constant parameter adjustments are needed, as the environmental conditions, print duration, and heat from motors can influence the end results. With larger sample sizes or tissue surrogates to be printed, ensuring and assessing cell viability and differentiation becomes difficult. Moreover, maintaining tissue viability and input of specific stimuli for proper maturation is still an ongoing challenge that will be decisive for the affirmation of the field. The general hindrances for the entire process are as follows: time, cost, sterility in all processes, and general reproducibility.

Ultimately, bioprinting is a highly multidisciplinary field requiring a broad, adaptable skillset. Researchers need to master the simultaneous handling of materials, technology, and biological design while keeping up with a rapidly evolving field. They need to succeed in creating printable and cell‐friendly materials to develop and use technology effectively while incorporating biological complexity into the designs.

To our knowledge and experience, the best approach is to embrace an interdisciplinary mindset from the beginning. Learning to communicate across biology, chemistry, and engineering is a prerequisite to bridge these disciplines, allowing all useful technical knowledge to converge into the work.

### 5.2 Technical Perspective

Future perspectives of where the field is heading are the direct answers to the mentioned limitations. Ideal bioinks need to be easily produced without high costs and be adaptable to both printing and, importantly, the following maturation processes. New dynamic materials that adapt and guide cell fate after printing are needed in the future. These materials not only need to be processable but also have to withstand and facilitate the remodeling induced by the cells by controlling and tuning degradation. Furthermore, spatially organized physical and chemical signals are needed to guide cell organization or binding, while the timely release of molecules or drugs from the inks themselves facilitates implantation, decreases inflammation, and prevents host rejection.

Bioprinting techniques are evolving, with hybrid approaches combining the advantages of different methods. The latest trends show an increase in contact‐free technologies to avoid mechanically harming cells. Multiple research workshops are offered around the world to build and use new printers and facilitate the transition from commercial to customized printers. Custom‐built printers can be tailored in specifications, from the number of degrees of freedom to multimaterial printheads to print plates accommodating large volumes, and offer far more flexibility, versatility and robustness toward the path of optimized organ‐specific printers. However, such printers require technical skills to set up, operate and trouble shoot.

Notably, how much should a bioprinted organ imitate a natural organ, which has evolved over millions of years, in terms of geometry and function Would it not be enough to produce an analog construct that functions as well as the native one. With respect to the cell component, one of the biggest bottlenecks to resolve is cell expansion to achieve billions and more required for organ printing. The use of stem cells has also been beneficial, as they play a fundamental role in proper human tissue development. With the differentiation of a patient's own cells, it will be possible to minimize the risk of rejection by the immune system.

### 5.3 Overall Perspective

Process optimization in the optics of scalability is one important step, and as discussed, simulations and predictive algorithms help optimize the process and increase productivity. Fundamental is also the standardization of practices across labs, making reproducibility and comparison possible. In this sense, regulatory agencies could be interesting partners in the field of bioprinting. The organs for regulatory affairs will certainly need to adapt the current standards and definitions to the new technology. The bioprinted tissues need to comply with quality assessments and good manufacturing practices to be in line with the level of excellency required for medical products to ensure their successful translation to a clinical practice standard.

Moreover, the printed functional tissues we developed thus far are already in use for multiple applications that are still very relevant for society, such as models for studying diseases, developing drugs, or testing cosmetics or chemicals for safety in the optics of Replacing, Reducing and Refining animal testing. On the one hand, these applications are already employed on an industrial level where incentivized by ever more strict governmental laws, such as the European ban on animal testing for cosmetics (Regulation EC 1223/2009), the need for better, animal‐free, and cheaper alternatives allows for the fast development of these platforms. On the other hand, biofabrication should also look closely at the developments of the medical field and the directions it is taking, find a path to intersect it and even take lead in directing progress as a perfect fit from the future perspective of personalized, predictive and preventive medicine.^[^ ^486^ ^]^

The field is piquing the interest of and recruiting new scientists from many fields, who want to employ technology to advance their own scientific questions. The broad distribution of the most recent technologies to experts in biology will help in revealing many biological mechanisms at the basis of tissue development. Even just thinking about how to design and print these artificial organs can offer valuable insight into better understanding the intricate interactions, structures, and development of tissues.

Only in the past 5 years the field has developed incredible technologies, e.g., volumetric bioprinting, sound‐based printing in terms of resolution, speed and versatility that were not imaginable before, unlocking new untapped potential to enhance and accelerate tissue bioprinting. Many of the challenges of yesterday are currently optimized parts of the workflow, and faster optimization, parallel to the advancement of new technological paradigms, makes it difficult to predict a timepoint for making bioprinting a reality.

With many people believing in the field and the attention from all sides, it is now up to the biofabricators to see the best way toward progress by interconnecting the knowledge from all fields and proving that what was thought to be fiction can be a reality.

## Conflict of Interest

The authors declare no conflict of interest.

## Supporting information



Supporting Information
