# Supplementary material for: Bioprinting Organs—Science or Fiction?—A Review From Students to Students
Source: Adv Healthc Mater. 2025 Dec 3;15(16):e02103. doi: 10.1002/adhm.202502103 (PMC13107929; doi:10.1002/adhm.202502103)
Supplement: Supplementary file 1 — Supporting Information [file ADHM-15-0-s001.pdf]

Supporting Information

Bioprinting organs - Science or fiction? - a Review from Students to Students

Nicoletta Murenu#, Camilla Mussoni#, Mateo S. Andrade Mier, Paula Buettner, Nathaly Chicaiza-Cabezas, Yi-Yu Robin Dai, Jessica Faber, Maren Fiedler, Zan Lamberger, Xuen Jen Ng, Vanessa Moessler, Anna Rederer, Jonas Roeder, Sabrina Stecher, Katinka Theis, Silvia Budday+, Gregor Lang+ and Natascha Schaefer+*

+shared last - These authors contributed equally to this work

#shared first - These authors contributed equally to this work

Supplement 1:

Survey- Printing Organs - Science or Fiction?

Biofabrication is an interdisciplinary science that deals with, among other things, the artificial production of tissue models or even whole organs. One branch of research focuses on using 3D bioprinting techniques to print living cells with hydrogels in a way that creates functional tissue. The following questions aim to capture an opinion on the potential of these technologies.
**We aim to understand a general sentiment on the topic presented. We will not collect any traceable personal information and only aggregate answer will possibly be used for the production of one publication.*

1. PreferredLanguage/BevorzugteSprache*

o English
o Deutsch (german version not provided here)

Survey in English

Welcome to the survey.
Thank you for taking a few minutes for this.

2. What is your level of knowledge about bioprinting or organ printing?*

o I have never heard of it
o I just heard of it
o I have read about it
o I work in this field myself

3. How important do you consider the topic of organ replacement in the societal context?*

	1	2	3	4	5	6	7	8	9	10	
**Not Important**	○	○	○	○	○	○	○	○	○	○	**Very Important**

4. Do you think that 3D printing organs is right?*

	1	2	3	4	5	6	7	8	9	10	
**No**	○	○	○	○	○	○	○	○	○	○	**Yes**

5. Do you want to tell us why you think so? *(not mandatory)*

6. How realistic do you think the following idea is: manufacturing artificial tissue that is equivalent to natural tissue using 3D printing

	1	2	3	4	5	6	7	8	9	10	
**Not Realistic**	○	○	○	○	○	○	○	○	○	○	**Very Realistic**

7. Where do you see the greatest challenge in organ printing? *(multiple choices possible)**

o Suitable materials that can be implanted without risk
o Precision of the technology
o Combination of different cells in one tissue/organ
o Keeping the artificial tissue alive and functional after printing
o Sufficient stability of the printed constructs to be implanted
o I don’t know

8.In which area do you see the greatest potential of bioprinting? *(multiple choices possible)**

o Food (artificial meat)
o Pharmacy (testing drugs or cosmetics on tissue models instead of animal)
o Organ replacement (printing parts of organs or entire organs as an alternative to organ donors)

9. Do you think it will be possible to print complete organs someday?*

o No, I think that's science fiction
o No, but I think parts of organs can be printed
o Yes, I think it will be possible to print and implant organs someday

10. When do you think organ printing will be a clinically established method?*

o Less than 10 years
o 10-20 years
o Longer than 20 years
o Never

Demographic

**We aim to understand a general sentiment on the topic presented. We will not collect any traceable personal information, and only aggregate answer will possibly be used for the production of one publication.*

11. If you work in science, what is your scientific background?*

o I don't work in natural sciences
o Medicine
o Life Sciences (Biology, Biochemistry...)
o Material Sciences / Chemistry
o Engineering
o Physics/Mathematics

12. How old are you?*

o <18
o 18-30
o 31-45
o 46-60
o >60

We thank you for your participation. If you have any suggestions or comments regarding this survey, we welcome your feedback:

____

Figure S2

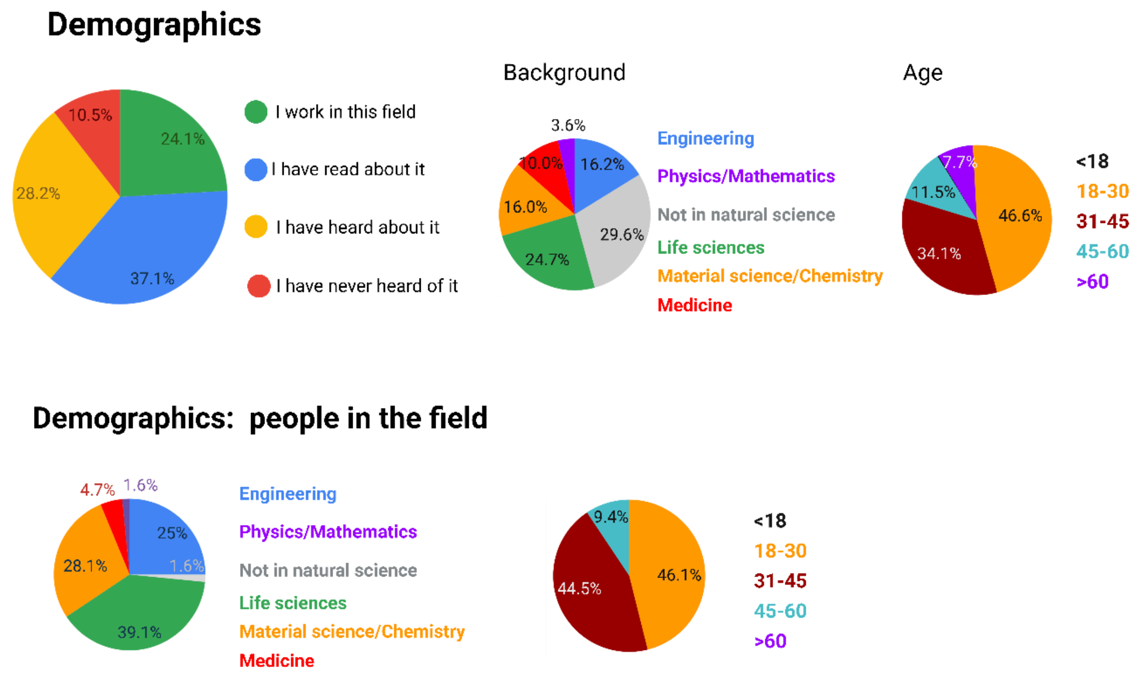

Figure S1. Demographics of the survey participants by level of expertise, background and age. Analysis of background and age of only of the expert of the field.
